# Expandable Nanocomposite Shape‐Memory Hemostat for the Treatment of Noncompressible Hemorrhage

**DOI:** 10.1002/advs.202508439

**Published:** 2026-02-06

**Authors:** Saptarshi Biswas, Sarah E. Miller, Shounak Roy, Jeevika Thazhaiselvam, Samantha Foster, Manivannan Sivaperuman Kalairaj, Sasha M. George, Yava Jones‐Hall, Staci J. Horn, Fred J. Clubb, Taylor H. Ware, Duncan J. Maitland, Akhilesh K. Gaharwar

**Affiliations:** ^1^ Department of Biomedical Engineering College of Engineering Texas A&M University College Station Texas USA; ^2^ College of Veterinary Medicine and Biomedical Sciences College Station Texas A&M University College Station Texas USA; ^3^ Interdisciplinary Program in Genetics College Station Texas A&M University College Station Texas USA; ^4^ Department of Material Science and Engineering College of Engineering College Station Texas A&M University College Station Texas USA; ^5^ Department of Veterinary Pathobiology College Station Texas A&M University College Station Texas USA; ^6^ Center For Remote Health Technologies and Systems College Station Texas A&M University College Station Texas USA

**Keywords:** biomaterials, hemostasis, nanocomposite, noncompressible hemorrhage, shape memory

## Abstract

Noncompressible hemorrhage is a leading cause of death in both combat and lay settings, primarily due to the challenges in accessing and treating injuries deep within abdominal tissues where traditional compression‐based methods are ineffective. Existing hemostatic materials fail to address the needs of large wound cavities, necessitating the development of advanced materials with rapid expansion and superior hemostatic properties. Here we report an expandable hemostat engineered from a nanocomposite‐coated shape memory foam. This material exhibits rapid expansion, achieving a ~5X increase in volume within 3 min in vitro, and demonstrates notable shape recovery in vivo. The material's hemostatic efficacy is evident through a ~70% reduction in clotting time compared to untreated controls, primarily due to the ability to adhere platelets and red blood cells. Moreover, the composite material displays excellent hemocompatibility and cytocompatibility, with low hemolysis rates and high cellular viability. In vivo assessments further confirm its effectiveness, showing an accelerated clotting time (~80% reduction) and decreased blood loss (~50% decrease), alongside minimal inflammation and necrosis in histological analyses. Additionally, the composite demonstrates good biocompatibility following subcutaneous implantation, illustrating its efficacy in vivo. Overall, the synergistic effect of rapid expansion by shape memory foam, along with the excellent procoagulant ability of the nanocomposite makes this biocompatible, expandable hemostat a promising treatment for noncompressible hemorrhage.

## Graphical abstract

A nanocomposite‐coated shape memory foam rapidly expands, accelerates clotting, and reduces blood loss in vivo. Its fast volumetric expansion and strong procoagulant activity make it a promising hemostat for treating deep, noncompressible hemorrhage.

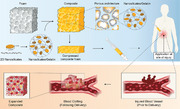

## 1 Introduction

Uncontrolled hemorrhage contributes to the majority of deaths in battlefield settings (~80% of casualties) and to ~40% of traumatic deaths in civilian settings due to massive blood loss, development of hypovolemic shock, and subsequent multiorgan failure [1, 2, 3, 4]. Noncompressible hemorrhage occurs in the truncal and junctional areas of the body and contributes to a staggering ~67% and ~53% of preventable deaths in truncal and internal injuries, respectively [5, 6, 7]. Noncompressible hemorrhage is untreatable with conventional hemostatic materials, as these injuries are not amenable to applying manual, external pressure [8, 9]. A range of engineered biomaterials has been developed as hemostats, including hydrogels, foams, sponges, and powders [10, 11]. However, due to the inability to access deep body wounds, conventional gauze or sponges, such as kaolin‐coated Combat Gauze or gelatin‐based Surgifoam, are not suitable for noncompressible hemorrhage [12, 13]. Conversely, hydrogels or powders, such as Celox, do not maintain their stability under high blood pressure and risk embolization if dissolved in the blood [4, 5, 14, 15, 16, 17, 18]. The ideal hemostatic material for treating noncompressible hemorrhage must facilitate rapid absorption of blood plasma, high adhesion to blood cells, accelerated activation of coagulation factors, and easy delivery inside the wound cavity while possessing both biocompatibility and biodegradability [9]. Additionally, these hemostats must be cost‐efficient, easy to fabricate, and shelf‐stable to increase their potential to be applied in diverse military and civilian environments [4, 10, 19]. These unmet requirements raise the need to develop an effective hemostat to treat noncompressible hemorrhage.

Shape memory biomaterials have gained significant attention as potential hemostatic materials for managing deep or internal injuries, including usage as embolization agents [20, 21, 22, 23] and for the treatment of traumatic hemorrhage [24, 25, 26, 27]. These materials display notable expansion from a compressed, secondary shape when heated due to segment relaxation above the foam's glass transition temperature [28]. In the presence of blood plasma, water‐driven plasticization of the polymer's bonds occurs to allow this expansion to unfold at body temperature [28]. The biodegradable nature of these foams allows for clinical improvement over other systems, which require careful surgical removal [29, 30, 31, 32, 33]. Additionally, these porous foams possess an extensive internal surface area, which in turn enables the foam to rapidly expand and absorb blood for accelerated hemostasis [24, 25]. Several research groups have designed hemostatic materials based on shape memory polymers, which are characterized by high expansion rates [25, 31, 34, 35, 36]. This expansion allows the material to exert pressure against bleeding vessels while simultaneously forming a physical plug. These constructs rely on the extensive surface area within the foam's architecture to trigger blood clotting, but do not contain any procoagulant ability within their structure and display limited adhesion to red blood cells [25, 37, 38, 39]. As a result, additional modification in the form of altering the chemical structure of the foam to incorporate additional molecules or providing bioactive coating is needed [37, 40].

Herein, we report an expandable hemostat engineered from a nanocomposite‐coated shape‐memory foam. We hypothesize that the rapid volumetric recovery of the shape‐memory polymer [24], together with the procoagulant activity of 2D nanosilicates [41, 42], will synergistically accelerate clot formation. Synthetic 2D silicate nanoparticles (~20–50 nm) possess anisotropic surface charges that promote clotting factor's activation [42] and can also serve as carriers for therapeutic loading [43, 44]. Gelatin, a denatured collagen derivative, enhances platelet aggregation and activation while remaining biocompatible and biodegradable [45, 46, 47]. Its heterogeneous charge distribution enables ionic stabilization of the nanocomposite and facilitates electrostatic interactions with red blood cells [27, 41, 51, 52]. Incorporating gelatin into the nanosilicate formulation, therefore provides complementary bioactivity and structural features conducive to rapid hemostasis.

Building on prior work [51], the shape memory hemostat increases the effective blood‐contacting surface area of the foam by introducing an optimized nanosilicate–gelatin coating that promotes rapid clotting and efficient fluid uptake. This surface modification enhances blood–material interactions without altering the expansion kinetics of the shape‐memory foam, thereby preserving its ability to absorb plasma rapidly. The nanocomposite coating further accelerates hemostasis by activating coagulation pathways and promoting platelet and red blood cell aggregation, yielding improved performance relative to the uncoated foam. In addition, the nanosilicate phase provides a platform for therapeutic incorporation, enabling potential wound‐healing or antimicrobial functionality. Following physicochemical characterization, we evaluated the efficacy of this expandable composite hemostat for managing noncompressible hemorrhage under both in vitro and in vivo conditions.

## 2 Results and Discussion

### 2.1 Fabrication and Characterization of Rapidly Expandable Nanocomposite Shape Memory Hemostat

**FIGURE 1:**
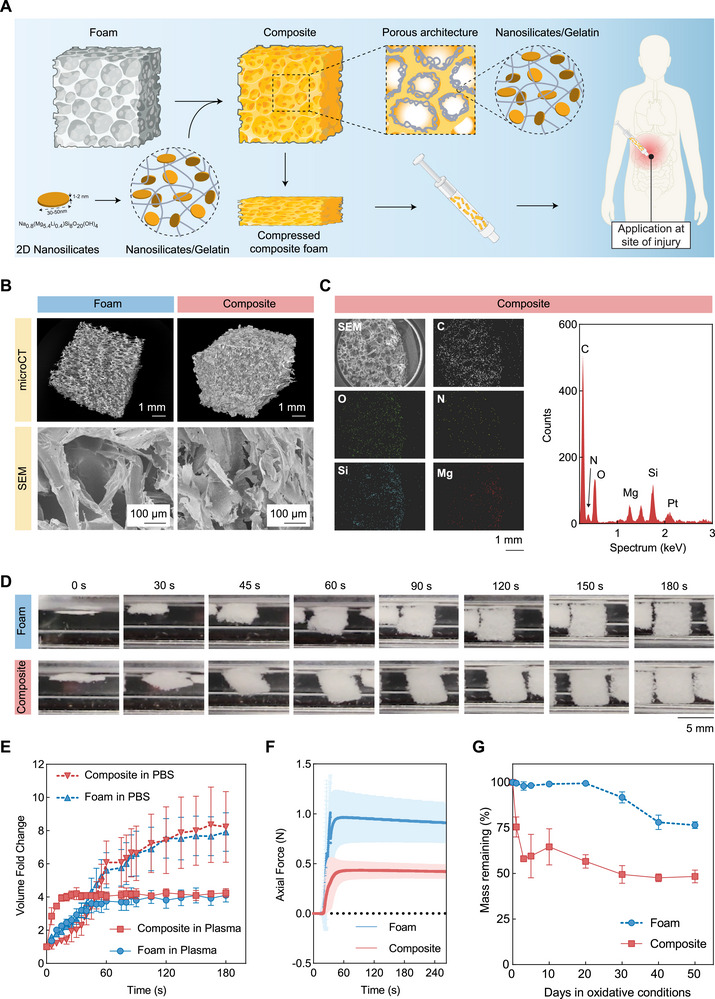
Characterization of microporous structure and rapid expansion ability. (A) Schematic representation showing the composite consisting of shape memory foam and nanocomposite, created partially using Biorender.com. (B) Macroscale and microscale (micro‐computed tomography; microCT and scanning electron microscopy; SEM, respectively) images of foam and composite highlighting macro‐ and microporous structures. (C) Energy dispersive X‐ray spectroscopy (EDS) of the composite sample (sputter coated with platinum‐palladium) and EDS spectrum indicating nanosilicate presence within the composite's porous structure. (D) Representative volume change of foam and composite at 37°C while immersed in phosphate‐buffered saline (PBS). (E) Volume fold change of foam and composite at 37°C in PBS and citrated human plasma. *N* = 3; data represented as mean ± standard deviation. (F) Axial force measurement of foam and composite at 37°C in PBS. *N* = 3; data represented as mean ± standard deviation. (G) Accelerated oxidative degradation study in 20% hydrogen peroxide for foam and composite, showing mass loss over time. *N* = 3; data represented as mean ± standard deviation.

We have designed the shape memory hemostat by internally and externally coating a porous foam with a nanocomposite (Figure 1A). The formulation of this nanocomposite was optimized based on the relative ratios of nanosilicate and gelatin present within the nanocomposite as well as the overall mass percentage (Figures S1–S5). In our previous work, we identified a trade‐off between fluid uptake and hemostatic ability [51]. We noted that a higher nanocomposite concentration yielded significantly improved fluid uptake, but hemostatic ability did not extend past this absorbed blood [51]. Conversely, the lower nanocomposite concentration resulted in poor fluid uptake, but a stronger ability to induce clotting in a volume of blood [51]. We attribute this improved hemostatic ability to enhance surface coating of the nanocomposite within the foam's pores and the subsequent increase in surface area available to activate coagulation and aggregate blood cells [51]. Thus, we sought to select a nanocomposite composition which balanced the need for a thorough surface coating while maintaining high fluid uptake. We identified a nanocomposite with 2% w/v nanosilicate and 1% w/v gelatin as the optimization point for coating the entirety of the foam's internal structure (Figures S2,S4, S5), as well as the ability to accelerate clotting (Figure S3).

In addition to selecting the nanocomposite composition based on our previous understanding of the impact of the overall mass percentage [51], we also explored the impact of the ratio between gelatin and nanosilicate in this nanocomposite coating (Figures S1–S4) to ensure the highest efficacy of our coating. The macroscale porosity of the foam and composites was observed using micro‐scale computed tomography (microCT) (Figure 1B), which displayed a highly interconnected porous structure in the foam. This porous structure was then filled with the nanocomposite to form the composite hemostat, which subsequently presented a microscale porosity seen in scanning electron microscopy images (SEM) (Figure 1B). The presence of nanosilicates within the composite's internal structure was confirmed by energy‐dispersive X‐ray spectroscopy (EDS) (Figure 1C). Nanosilicates were present throughout the structure, indicated by the presence of silicon and magnesium in both edge and central regions of the composite (Figure S5), though the majority of the nanocomposite appeared closer to the external surface of the sample (Figure 1C; Figure S5). We hypothesized that this accumulation of nanocomposite on the edge of the composite structure as well as thorough penetration into the foam would result in the desired combination of fluid uptake and hemostatic ability. Together, these results demonstrate the successful incorporation of the nanocomposite within the shape‐memory polymer foam. Based on these promising results, we identified a composite consisting of a nanocomposite with 2% w/v nanosilicate and 1% w/v gelatin for further experiments.

The shape‐memory polymer foam component in the composite hemostat is well known for its rapid expansion ability when exposed to fluids at or above body temperature [24, 36]. Both foam and composite demonstrated rapid expansion in PBS within 180 s, retaining their original shape, which could be crucial to absorbing wound exudate at the site of injury to promote the healing response (Figure 1D). The expansion rate and force were characterized to ensure that the expansion property was retained in the composite formulation. The expansion rate was noted to be nearly identical between the foam and composite in both PBS and citrated blood plasma (Figure 1E). A slight decrease in the overall expansion ability of the samples was noted between expansion in PBS versus expansion in blood plasma. This phenomenon is likely attributed to proteins and small molecules present in blood plasma, which increase the viscosity of blood plasma relative to PBS and therefore limits the plasma's ability to infiltrate the internal structure of the sample. Additionally, the composite displayed decreased expansion force compared to the foam alone (Figure 1F), which is likely due to the interconnected and microporous network present within the composite, thus limiting the impact of expansion. However, both formulations of samples were observed to expand to their final dimensions within 3 min, suggesting a high potential for use in the rapid filling of wound cavities. These findings are similar to results seen in our previous work [51]. The swelling percentage was also evaluated for both compressed foam and composite, where, due to the foam's extremely low dry weight, the swelling percentage of foam was found to be higher compared to the composite (Figure S6A). However, the fluid absorbed per sample was higher in the composite than in the foam, especially over shorter time points (Figure S6B). Although this increase was not statistically significant, it demonstrates an improvement over our previous infused composite [51]. Further, a remarkable initial spike of fluid uptake was confirmed for both experimental groups, indicating its potential for rapid plasma absorption at the wound cavity for rendering hemostasis (Figure S6A,B).

The degradation profiles of both foam and composites were assessed under accelerated oxidative, hydrolytic, and enzymatic conditions. Under oxidative conditions, within the initial 20 days, a significant mass decrease was not observed for the foam, but after 20 days, a slow mass decrease was observed, finally reaching a remaining mass of ~75% at 50 days. Alternatively, the composite demonstrated an initial sharp mass loss, which is followed by minimal mass loss thereafter, and became stabilized after 30 days with an overall ~50% decrease in mass (Figure 1G). The same trend has been observed with varying concentrations of H_2_O_2_ solutions and different pH, where no mass loss of foam was obtained after 21 days, but composite showed mass loss in all conditions (Figure S7A–F). This could be due to the rapid dissolution of the nanocomposite, containing nanosilicate and gelatin, from the composite, exposing the native foam structure in the oxidative environment at a later time point. Additionally, the physical crosslinking and hydrophilic interaction between nanosilicate and gelatin lose their stability at 37°C, resulting in the diffusion of the nanocomposite from the composite. Our previous study has reported minimal mass loss of foam until 54 days in prolonged oxidative conditions. However, the mass loss of foam has increased significantly after 54 days, which is attributed to the fragmentation of the foam structure after initial surface degradation [24, 51]. In another study, for shape memory polymer foam, it has been found that under oxidative conditions, the C‐N bond of a tertiary amine degrades to lower amines, aldehydes, and carboxylic acids [24, 52]. Additionally, rapid degradation of the coated composite was found to be 18 days earlier than the native foam, which can be extrapolated to ~260 days early in vivo conditions, suggesting that our composite can degrade within approximately 3‐4 years in vivo and be competitive with other biodegradable foams [24, 51, 52]. Apart from modifying the chemical structure of the foam, these results show that the degradation rates of the composite can be modified by coating the foam with a hydrophilic nanocomposite to draw more water molecules and reactive oxygen species, leading to the potential of developing hemostats with enhanced biodegradation ability. An accelerated hydrolytic and enzymatic degradation study was performed accordingly, where an initial rapid mass loss was observed for the composite, but no observable change in the mass of foam was seen, indicating the ability of the composite to degrade rapidly (Figure S7G,H).

During degradation, the nanocomposite components, containing nanosilicate and gelatin are expected to degrade or dissolve at earlier timepoints under physiological temperature, as gelatin melts around 37°C, and nanosilicate has been found to dissociate in acidic and physiological pH. During our degradation studies, we observed sedimentation of the nanocomposite in the degradation solution, and this particulate matter remained after evaporating off the degradation solution, which was not observed for the equivalent foam‐only samples. An earlier study showed that dissociation of nanosilicate under acidic conditions [53]. Pang et al. reported an additional FTIR peak at 1734 cm^−1^ appears under oxidative and enzymatic degradation of gelatin‐hydrogel that could occur due to the expected cleavage of ester or amide bonds of gelatin, forming ‐COOH terminated chains [53]. Previous studies have also shown that the lysine, arginine, and proline of gelatin undergo oxidative degradation to produce small molecules, e.g., α‐ketoacid, carboxylic acid, ammonium ion, oximes, and carbon‐dioxide [54, 55, 56].

Regarding degradation of shape memory foams, the rate of degradation is correlated with the ester content since under oxidative condition, the tertiary amines of TEA and HPED oxidize to produce carboxylic acid, aldehydes, and primary amines [52]. Jang et al. reported a carbonyl peak shift from 1689 to 1696 cm^−1^ of TEA‐containing foam, confirming C‐N bond fragmentation to form aldehydes and aldehyde conjugates. Additionally, a small decrease in the urea peak is also expected for foam, especially with higher ester content. The group also observed surface degradation of control foams for 90 days, whereas ester‐containing foams showed bulk degradation. This occurrence could be attributed to the fact that the reactive hydroxyl radical, produced from H_2_O_2_, is unstable and causes chain scission of tertiary amines on the surface [24, 57, 58]. Taken together, at later timepoints, degradation of H40 foam presented in this study could lead to formation of aldehydes, carboxylic acids and primary amines, whereas degradation of hydrogel part of the composite is expected to produce α‐ketoacid, carboxylic acid, nontoxic Na^+^, Li^+^, Si(OH)_4_, and Mg^2+^, which can undergo continuous degradation and clearance inside the body [59].

### 2.2 Expandable Composite Demonstrates Rapid In Vitro Hemostasis

**FIGURE 2:**
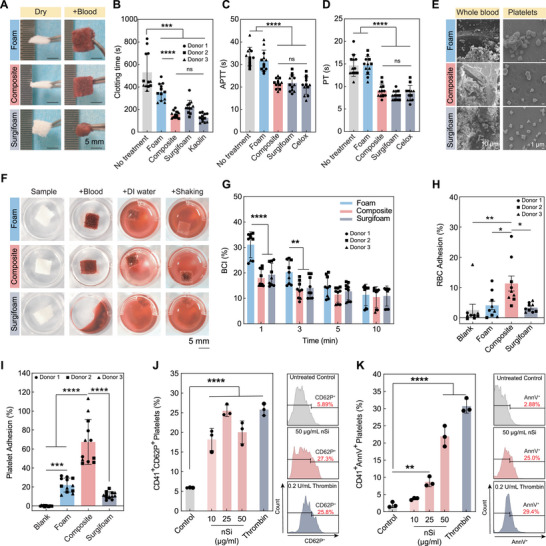
In vitro hemostatic ability of composite. (A) Photographs of foam, composite, and Surgifoam before (Dry) and after (+Blood) exposure to whole rat blood at body temperature, illustrating expansion of foam and composite and shrinkage of Surgifoam. (B) Quantitative clotting time measurement determined via the inversion test. Positive controls: Surgifoam and kaolin; negative control: no treatment. *N* = 12 with four replicates from each of the 3 biological replicates. Data represented as mean ± standard deviation. (C) APTT measurement. Positive controls: Surgifoam and Celox; negative control: no treatment. *N* = 12 with 4 replicates from each of the 3 biological replicates. Data represented as mean ± standard deviation. (D) PT measurement. Positive controls: Surgifoam and Celox; negative control: no treatment. *N* = 12 with 4 replicates from each of the 3 biological replicates. Data represented as mean ± standard deviation. (E) SEM images of foam, composite, and Surgifoam, illustrating red blood cell adhesion and clot formation (left column) and platelet adhesion and activation (right column). Scale bars are 10 µm (left column) and 1 µm (right column). All images were adjusted by −20% brightness and 100% contrast using Photoshop to aid in visual clarity. (F) Representative images of blood clotting index (BCI) samples. (G) Quantitative measurement of BCI. Positive control: Surgifoam. *N* = 9 with 3 replicates from each of the 3 biological replicates. Data represented as mean ± standard deviation. Statistical test performed was a two‐way ANOVA with Tukey's post hoc test. (H) Red blood cell adhesion. Positive control: Surgifoam; negative control: blank tubes. N = 9 with 3 replicates from each of the 3 biological replicates. Data represented as mean ± standard error. (I) Platelet adhesion. Positive control: Surgifoam; negative control: blank tubes. N = 9 with 3 replicates from each of the 3 biological replicates. Data represented as mean ± standard error. (J) Quantitative analysis showing the percentages of CD41^+^ CD62P^+^ cells. Data represented as mean ± standard deviation. Representative histogram showing the expression of CD62P on the surface of control, 50 µg/mL nanosilicate, Thrombin‐ treated CD41^+^ platelets, as measured by flow cytometry. (K) Quantitative analysis showing the percentages of CD41^+^ AnnV^+^ cells. Data represented as mean ± standard deviation. Representative histogram showing the externalization of phosphatidylserine on the surface of control, 50 µg/mL nanosilicate, thrombin‐treated CD41^+^ platelets, as measured by Annexin V‐FITC flow cytometry. *Note*: Data points from each biological replicate are identified via circles (replicate/donor 1), squares (replicate/donor 2), and triangles (replicate/donor 3). The individual donors in each experiment do not necessarily correspond to the same donors in other experiments.

The uptake of blood into the samples was explored visually using macroscale photographs. The compressed foams and composites were observed to expand into a larger shape upon absorbing blood (Figure 2A), while the Surgifoam sample did not demonstrate any expansion, as expected. Of note, Surgifoam appeared to condense and shrink upon contact with blood, likely due to the gelatin structure melting and dissolving in the presence of body‐temperature blood. Hemostatic ability was tested in vitro via whole blood clotting time, using an inversion method with recalcified whole human blood [4]. All the samples were able to clot blood eventually. Without any treatment, the clotting time was found to be ~ 531 ± 168 s, whereas, in the presence of foam and composite, the clotting time was significantly reduced. This is likely because both foam and composite were able to expand rapidly, in the presence of blood, providing a high contact area and tortuous structure to aggregate blood cells. The composite's clotting time was similar to the values obtained from the positive controls, Surgifoam and kaolin (Figure 2B). This is consistent with our previous studies, wherein the presence of nanosilicate and gelatin‐containing hydrogel in the composite resulted in a further decrease in the clotting time [41]. Using a similar procedure, the whole blood clotting test was also performed with recalcified whole bovine blood, where the findings followed a similar trend as described above. The clotting time of the composite was found to be ~143 ± 26 s, which is significantly reduced compared to both no treatment and foam, resulting in ~78% reduction compared to the no treatment value (Figure S8A).

Secondary hemostasis is comprised of the intrinsic and extrinsic coagulation pathways, which lead into the common pathway for fibrin formation [60]. The effects of foam and composite on each of these pathways were evaluated by performing activated partial thromboplastin time (APTT) and prothrombin time (PT) assays. The APTT of the no treatment group and of foam indicated no significant effect of foam on the intrinsic pathway. In the presence of the composite, the APTT was significantly reduced, resulting in an almost 35% decrease compared to no treatment (Figure 2C). Previous studies have indicated that, owing to its negatively charged surface, silica can activate factor XII of the intrinsic pathway to trigger the cascading mechanism of intrinsic coagulation, which could validate the reduction of APTT for the composite [61, 62]. Similar to APTT values, the same trend has been observed in assessing the PT. Compared to the no‐treatment group, in the presence of the foam, the PT value did not decrease. However, when the composite was applied, the PT value was significantly reduced compared to the no treatment group, indicating around 38% reduction in PT value. The PT values in the presence of commercially available Surgifoam and Celox were comparable with the PT of the composite (Figure 2D). Due to the presence of silica in the nanocomposite coating in the composite, the expression of tissue factors (TF) could be increased along with the activation of exogenous coagulation factors, resulting in a decreased PT value [63, 64]. These results demonstrated that while formulating the composite structure, combining nanocomposite with the shape memory foam was crucial to activating both coagulation pathways.

The blood clotting index (BCI) provides another avenue to assess hemostatic ability by quantifying uncoagulated red blood cells through the absorbance of hemoglobin. Hence, a lower BCI indicates better procoagulant ability [34, 65]. Representative images of experimental groups are depicted in Figure 2E. After incubating the samples with whole human blood, the samples were washed to collect unbound blood cells and shaken to release hemoglobin from these unbound cells. Therefore, the color of the solution inversely represents blood‐material interaction, and a lighter color indicates better coagulation. At 1 min, the BCI of the composite was significantly less than the BCI of the foam, and was found to be similar to the BCI of Surgifoam, (Figure 2F). The BCI values of all experimental groups dropped at 3 min. Further, a similar trend of reduced BCI was observed for all experimental groups until 5 min, but the BCI of all experimental groups did not vary from the 5‐ to 10‐min time intervals. This result suggests better red blood cell adhesion on the composite surface compared to the foam within 1 min and was consistent till 3 min, which could be attributed to the fact that negatively charged red blood cells can adhere strongly to the positively charged edges of nanosilicate and to gelatin, demonstrating excellent procoagulant ability. Owing to the lack of nanosilicate‐gelatin nanocomposite in the foam, the adherence between the foam surface and red blood cells is compromised at early time points. However, the BCI values were found to be saturated after approximately 5 min. We also performed the BCI analysis with whole bovine blood using a similar protocol, where a similar result trend was observed as described above. The results indicated low BCI of the composite within 1 min, compared to foam, and the BCI continued to drop until 5 min and became saturated afterward, validating high red blood cell adhesion on the composite's surface in bovine blood as well (Figure S8B).

Additionally, blood clots were observed within the foam and composite's porous structures using SEM imaging. Imaging of internal cross‐sections of these samples revealed abundant red blood cells in each of the foam, composite, and Surgifoam materials (Figure 2G). Additionally, fibrin networks and aggregates of red blood cells were noted to be present within each of the materials. These observations are in line with the previous observations of the shape‐memory foam alone and its ability to sequester large volumes of blood within its structure and promote clot formation [24, 66].

Red blood cell adhesion to the material surface was explored further using a solution of isolated red blood cells. The composite demonstrated the highest adhesion of red blood cells (Figure 2H). This adhesion level was significantly increased compared to all other groups. Similarly, the composite presented the highest quantitative platelet adhesion observed using a solution of isolated platelets (Figure 2I). Again, this adhesion level of the composite was significantly increased compared to all other groups. These results can be attributed to a combination of the materials’ characteristic surface activity and the ability of the material to absorb fluid and retain cells: the composite presents both a macroporous structure as well as a microporous, adhesive structure due to the gelatin‐based nanocomposite coating within the macroporous foam and is able to retain fluid and cells following absorption. Further, the charged species present in the form of both ampholytic gelatin and nanosilicates enable ionic interactions with blood cells. While the foam does contain this macroporous structure, the relatively lower surface texture and activity in comparison to the composite render a decreased ability to retain adhered red blood cells and platelets. Although Surgifoam, as a gelatin sponge, presents abundant cell adhesion motifs, this structure melts in physiological temperature, which limits the material's ability to retain absorbed fluid and the lack of nanosilicate further reduces the interaction with blood cells. We observed the infiltration of red blood cells by incubating these materials in citrated whole blood and subsequently sectioning the materials to observe cells present in the internal structure. All materials presented aggregates of blood cells (Figure S8) with a qualitative increase in the composite, suggesting that the presence of nanosilicates impacted the cellular adhesion. These results highlight the synergistic effect of the nanocomposite and foam to produce an effective hemostatic material.

We further evaluated the potential of nanosilicates, present in our nanocomposite coating, to activate platelets. Platelets play an important role in regulating hemostasis. Once activated, platelets undergo changes in their morphology, adhere to the vessel walls, and secrete several potent signaling molecules, which support the coagulation cascade. One of the key biomarkers of platelet activation is the enhanced expression of the surface antigen, P‐Selectin (CD62p) on the surface of activated platelets [67]. We incubated freshly isolated platelets with nanosilicates and monitored the percentage of CD62p+ platelets using flow cytometry. The gating strategy and representative plots are provided in Figures S10 and S11, respectively. As shown in Figure 2J, we observed a significant increase in the percentage of CD62p^+^ platelet population in samples treated with different concentrations of nanosilicates, compared to non‐treated (unstimulated) platelets. Another key event associated with platelet activation is the translocation of phosphatidylserine (PS) from the inner leaflet to the outer leaflet of the platelet membrane. PS externalization transforms activated platelets into highly procoagulant surfaces that boost thrombin production and promote the formation of a stable fibrin clot [68]. We measured the extent of PS externalization on platelets treated with nanosilicates through the Annexin V‐FITC assay. Annexin V has a strong affinity for binding to PS and is extensively used as a probe to study PS externalization using flow cytometry. The gating strategy and representative plots are provided in Figures S12 and S13, respectively. Flow cytometric percentage of Annexin V‐FITC^+^ platelets was significantly higher in platelets treated with increasing concentrations of nanosilicates, when compared to unstimulated control. When treated with 25 and 50 µg/mL of nanosilicate, approximately 5X and 10X increase in percentage of Annexin V‐FITC^+^ platelets were observed respectively, compared to the control (Figure 2K). These results further support the SEM image of platelets adhered to the surface of the composite, which clearly shows “spiky” morphology of platelets with extended filopodia, indicating signs of activation. Overall, these results together demonstrate the platelet‐activating potential of nanosilicates present in the nanocomposite coating of the shape‐memory hemostat, thereby acting as a key component for ensuring rapid and stable hemostasis.

### 2.3 Expandable Composite Demonstrates Strong Hemocompatibility and Cytocompatibility

**FIGURE 3:**
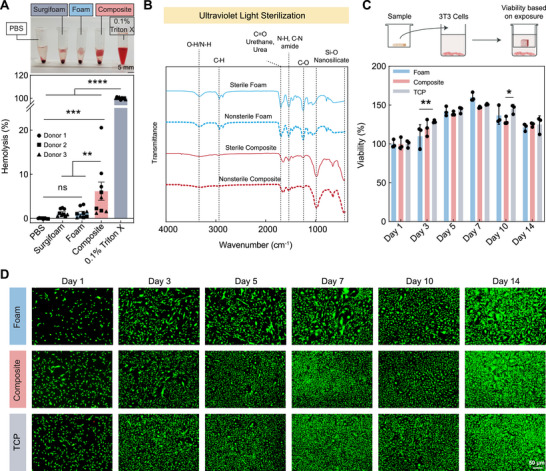
Hemo and cytocompatibility of hemostatic composite in vitro. (A) Hemolysis evaluation. Positive control: 0.1% Triton X; negative controls: Surgifoam and PBS. N = 9 with 3 replicates from each of the 3 biological replicates. Data represented as mean ± standard error. (B) FTIR analysis of foams and composites before and after sterilization, showing the presence of key chemical peaks. (C) Alamar Blue assay to quantify cellular viability following exposure to leachables from foam, composite on NIH 3T3 mouse fibroblasts. The schematic was created partially using Biorender.com. The values are normalized with day 1. N = 3; data represented as mean ± standard deviation. Statistical test performed was a two‐way ANOVA with Tukey's post hoc test. (D) Live/Dead assay to observe cellular morphology and viability following exposure to leachables from foam, composite, and tissue‐culture plate (TCP control) on NIH 3T3 mouse fibroblasts. Brightness of images has been enhanced uniformly using Adobe Photoshop to aid in visualization. Original images and Photoshop files are available in Zenodo repository.

In addition to blood clotting ability, hemocompatibility and cytocompatibility were evaluated via hemolysis testing, live/dead staining, and Alamar blue assay, respectively. Hemolysis of the foam and the composite was compared to PBS (negative control), Surgifoam (clinical control), and 0.1% Triton X (positive control) (Figure 3A). The hemolysis measurement was normalized to display 0.1% Triton X at 100% hemolysis. In this study, we observed that both Surgifoam and the foam alone displayed minor hemolysis, with average hemolysis at 1.27% ± 0.26% and 1.25% ± 0.37%, respectively. The composite sample demonstrated a higher level of hemolysis, at an average of 6.16% ± 2.06%, which falls just above the range for acceptable hemolysis levels [69]. This increase in hemolytic activity is attributed to the presence of nanosilicates. This observation further indicates that optimization of the nanocomposite is necessary to minimize the nanosilicate component while still achieving rapid clotting.

Sterilized foams and composites were also prepared and evaluated, prior to testing in cellular assays. Samples were sterilized using ultraviolet (UV) light exposure and subsequently evaluated using FTIR to determine if any chemical changes occurred. No change is noted between non‐sterilized and sterilized samples (Figure 3B). Foam samples display characteristic peaks for polyurethane foams. The foam's characteristic peaks are muted in the composite due to the presence of the nanocomposite on the surface of the sample. Large peaks for Si‐O bonds are observed in the composite sample, indicating the presence of nanosilicates. Therefore, the use of UV light sterilization is appropriate for use on these samples.

The cytotoxicity of the foam and composite was evaluated using the Live/Dead assay and Alamar Blue assay. Both assays were performed using NIH 3T3 mouse fibroblast cells over 14 days. The initial weight loss of the components of both foam and composite led to the accumulation of analytes in the media. Therefore, we wanted to check the effect of the leachables on cellular growth. An indirect contact transwell method was employed, where the cytotoxic effect of leachables from the samples was evaluated. Alamar Blue result indicated increased viability compared to day 1 in all groups, indicating cellular proliferation, which was maximum at day 7, approximately with 150%. Though after 7 days, a drop in viability was observed, the values were still higher than day 1, which is consistent in each group, attributing to the fact that cells had undergone slower proliferation after 7 days (Figure 3C). Live/Dead assay confirmed the presence of cells with distinct cellular borders, which grew over time in all groups. In comparison to TCP, no significant decrease in cell number was observed for both foam and composite in all time intervals (Figure 3D). These results indicate that the foam and composite are safe to be used before evaluating their efficacy in an in vivo system, as the hemostatic materials must possess minimum cytotoxicity during hemostasis after being delivered to the site of injury [70, 71]. In addition to innate cytocompatibility, we wanted to check whether we could load an antibacterial solution to incorporate antibacterial properties in the fabricated samples. When gentamicin was directly loaded on top of the samples, we observed that native foam and composite did not show any antibacterial properties, but gentamicin‐infused samples showed no bacterial growth after 24 h. Interestingly, when gentamicin was loaded into the nanocomposite, prior to fabricating gentamicin‐loaded composites, the composites demonstrated antibacterial properties for 3 days. Alternatively, foam showed antibacterial properties only for 1 day, which indicates the potential of the integrative strategy to fabricate composites with loaded therapeutics to exhibit antibacterial properties to prevent microbial infection at the site of injury for accelerated hemostasis and wound healing (Figure S14A,B). This loading and release of several therapeutics to augment the process of hemorrhage control and rapid wound healing will be explored in our future studies in further detail.

### 2.4 Expandable Composite Exhibits Accelerated Hemostasis and Reduced Blood Loss in Rat‐Liver Laceration Model

**FIGURE 4:**
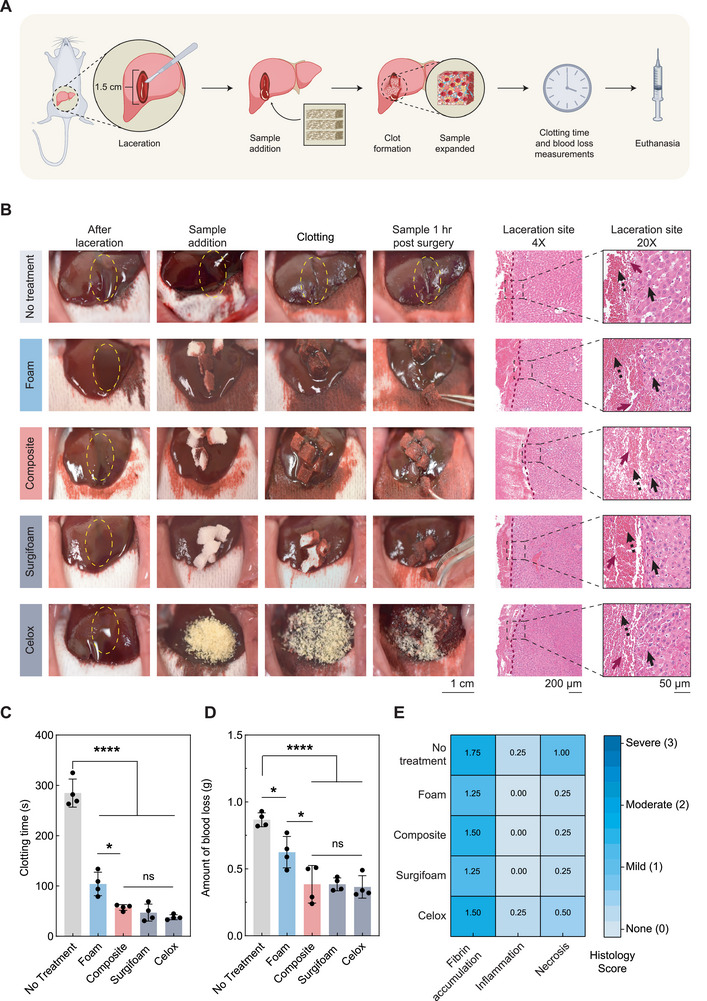
In vivo evaluation of expandable hemostatic composite in a rat liver laceration model (A) Surgery schematic of rat liver laceration model. Surgifoam and Celox serve as clinical positive controls, and no treatment serves as negative control. The schematic was created partially using Biorender.com. (B) Images of the lacerated liver, lacerated site after adding sample, lacerated site after clot formation, and 1 hour (hr/h) post‐surgery, as well as H&E images of the laceration site 1 h post‐surgery at 4X and 20X magnification. Surgifoam and Celox serve as clinical positive controls, and no treatment serves as negative control. The magenta solid arrow indicates fibrin, the black dotted arrow indicates RBC, and the black solid arrow indicates hepatocytes surrounding the biopsy punch site. Representative images of sample 1 h post‐surgery for the no treatment and Celox group are the same as the image of the laceration site 1 h post‐surgery for the no treatment and Celox group respectively in Figure S15. (C) Clotting time and (D) blood loss evaluation. Surgifoam and Celox serve as clinical positive controls, and no treatment serves as negative control. N = 4, biological replicates; data represented as mean ± standard deviation. (E) Heat map displaying subjective histology score from the pathologist. Surgifoam and Celox serve as clinical positive controls, and no treatment serves as a negative control. The score is for necrosis, inflammation, and fibrin localized around the laceration site as visualized in the histology of lacerated liver tissue, 1 h post‐surgery. N = 4, biological replicates; data represented as mean.

The composite's hemostatic ability was assessed in vivo using a rat liver laceration model wherein an approximately 1.5 cm laceration was performed in the left medial liver lobe (Figure 4A). The representative images indicate the expansion of samples upon delivery at the site of injury and the ability to seal the wound rapidly (Figure 4B). Both the foam and the composite maintained their expanded structure 1‐hour post‐surgery, and following the sample removal; the representative laceration site after 1 h of injury is presented in Figure S15. Following sample addition, clotting time and the amount of blood loss were quantified. Without any treatment, the clotting time was found to be ~285 ± 28 s, which, with application of foam, significantly decreased to ~104 ± 23 s. In the presence of the composite, the clotting time further decreased to ~57 ± 5 s, resulting in a remarkable ~80% decrease in clotting time compared to no treatment. When Surgifoam and Celox were added to the laceration site, the clotting time was calculated to be ~47 ± 17 s and ~38 ± 5 s, respectively, which is similar to the clotting time value obtained after composite addition (Figure 4C). The quantification of the amount of blood loss demonstrated a similar trend as clotting time, where no treatment resulted in blood loss of ~0.86 ± 0.05 g, but was significantly decreased by the foam (blood loss of ~0.62 ± 0.11 g). Following composite delivery, the blood loss further decreased to ~0.38 ± 0.14 g, resulting in a ~56% decrease in the amount of blood loss compared to no treatment (Figure 4D). Clotting time and blood loss were both significantly reduced for the composite, compared to no treatment (Figure 4C,D).

To investigate the effect of samples on the lacerated liver tissue, histological analysis was performed around the localized site of liver injury, and necrosis, inflammation, and fibrin deposition were assessed. The histological analysis exhibited both aggregated blood cells and fibrin strands at the site of injury in all experimental groups, consistent with our in vitro results. Further, fibrin accumulation at the injury sites validated the firmness of the formed clots, and the hepatocytes around the site of injury did not exhibit any notable change to their morphology (Figure 4B). Minimal necrosis and inflammation, along with moderate fibrin accumulation, were observed around the injury sites for all experimental groups, illustrating the safety of foam and composites to be delivered in vivo (Figure 4E).

### 2.5 Expandable Composite Demonstrates Rapid Hemostasis and Lower Blood Loss in a Noncompressible Rat‐Liver Biopsy Punch Model

**FIGURE 5:**
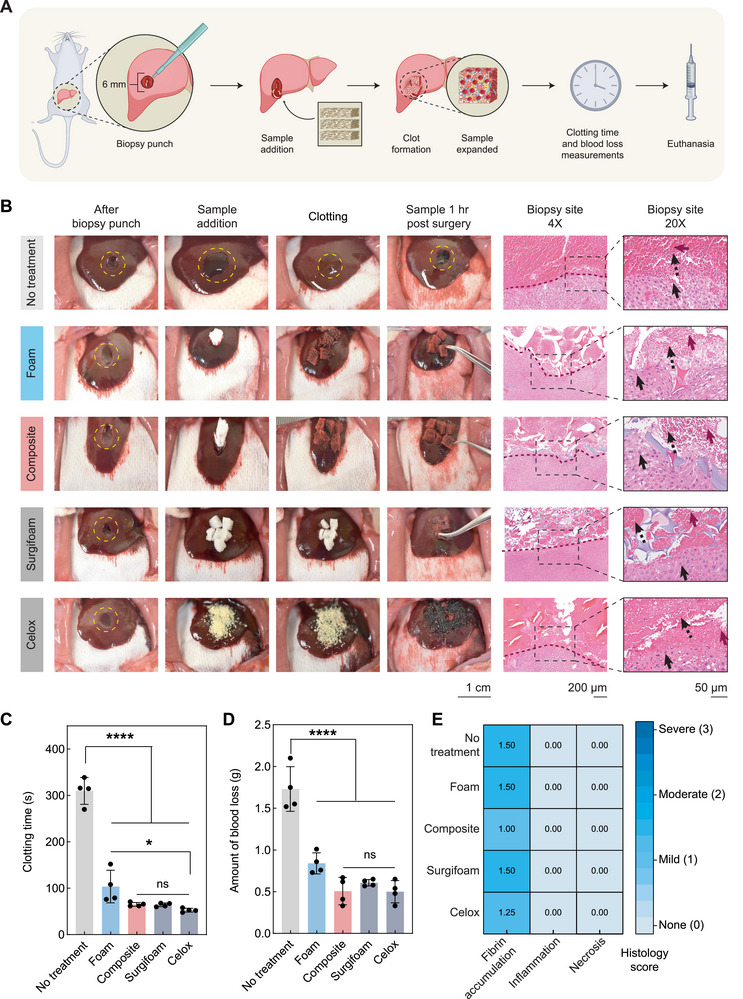
In vivo rat liver biopsy punch model to evaluate hemostatic efficacy of the expandable composite in noncompressible hemorrhage. (A) Surgery schematic of rat liver biopsy punch model. Surgifoam and Celox serve as clinical positive controls, and no treatment serves as negative control. The schematic was created partially using Biorender.com. (B) Images of the liver with a biopsy punch, injury site after adding sample, injury site after clot formation, and 1 hour (hr/h) post‐surgery, as well as H&E images of the injury site 1 h post‐surgery at 4X and 20X magnification. Surgifoam and Celox serve as clinical positive controls, and no treatment serves as negative control. The magenta solid arrow indicates fibrin, the black dotted arrow indicates RBC, and the black solid arrow indicates hepatocytes surrounding the laceration site. Representative image of sample 1 h post‐surgery for the no treatment and Celox group is the same as the image of the biopsy punch site 1 h post‐surgery for the no treatment and Celox group, respectively in Figure S15. (C) Clotting time and (D) blood loss evaluation. Surgifoam and Celox serve as clinical positive controls, and no treatment serves as negative control. N = 4, biological replicate; data represented as mean ± standard deviation. (E) Heat map displaying subjective histology score from the pathologist. Surgifoam and Celox serve as clinical positive controls, and no treatment serves as negative control. The score is for necrosis, inflammation, and fibrin localized around the injury site as visualized in the histology of injured liver tissue, 1 hour post‐surgery. N = 4, biological replicates; data represented as mean.

We further tested the composite's hemostatic efficacy using a noncompressible rat liver biopsy punch model (Figure 5A). An incomplete hole was created to produce a cavity in the liver, using a 6 mm biopsy punch, without fully puncturing the liver. After creating the injury, exactly one sample type was administered, and clotting time and blood loss were quantified. The wound area is filled due to the rapid expansion of foam and composite, forming a mechanical hemostatic plug to stop bleeding (Figure 5B), which was consistent even after 1 h of injury. Representative biopsy punch site is presented in Figure S15. Without any treatment, the clotting time was ~310 ± 29 s, which was significantly reduced in the case of treatment with either foam or composites. In the presence of foam, the clotting time was ~104 ± 35 s, while the clotting time for the composite group was decreased to ~65 ± 4 s, which was comparable with the clotting time obtained with both positive controls. The result suggests that the composite was able to reduce the clotting time by ~80%, even in a noncompressible hemorrhage model (Figure 5C). Though the composite did not present a statistically significant improvement over the foam alone, ~40 s reduction in clotting time has still been observed, which could be very important to establish the clinical significance of the composite to increase the “golden hour” for improving patient survivability. Interestingly, when foam and composite were analyzed separately, statistical significance was observed, which could be attributed to the fact that the high clotting time related to the no treatment group affected the overall statistical significance among the experimental groups (Figure S16A).

As the liver biopsy punch model mimics noncompressible hemorrhage in an internal body organ, where a pressure dressing is not feasible, we observed a ~2X increase in blood loss for untreated animals compared to the untreated animals in the liver laceration model. Without any treatment, the amount of blood loss was found to be ~1.72 ± 0.27 g, but upon delivery of foam and composite, the blood loss decreased significantly to ~0.84 ± 0.13 g and ~0.50 ± 0.16 g, respectively (Figure 5D). The composite demonstrated similar results to both positive controls, Surgifoam and Celox, which represented an approximately 70% reduction compared to no treatment. Despite no statistical significance, compared to foam, ~ 0.34 g blood loss reduction was observed in the presence of composite, indicating its potential for better clinical patient outcomes in treating noncompressible hemorrhage. This is consistent with the trend observed in the clotting time, when blood loss after applying foam and composite was analyzed separately, statistical significance was observed accordingly (Figure S16B). The composite samples were also evaluated in an ex vivo porcine liver biopsy punch (Video S1). The composites were injected into a cavity created using a 6‐mm biopsy punch and demonstrated approximately 3X volume expansion to fill the space rapidly. This indicates the potential efficacy of the composite to treat noncompressible hemorrhage in the large animal models as well.

Following histological analysis, we confirmed that fibrin and blood cells were accumulated at the injury site, contributing to the firmness of the thrombus formed during hemostasis. Additionally, the structure and integrity of the hepatocytes remained intact, indicating that the interaction between samples and cells was not toxic (Figure 5B). Assessment of the obtained histological score confirmed minimal necrosis and inflammation along with moderate fibrin deposition in all experimental groups (Figure 5E).

Owing to its high expansion property, the composites were able to absorb blood rapidly, and the presence of nanosilicate‐gelatin nanocomposite on the surface was crucial for red blood cells and platelets to aggregate and adhere to the material. This conclusion is supported by previous studies, which have reported that nanosilicate can activate platelets and coagulation cascades and that gelatin exhibits high adhesion due to an abundance of cell attachment motifs [72, 73]. Further, the relationship between hemostatic ability and material porosity and surface area was explored. MicroCT was performed on each of the four sample groups (i.e., foam, composite, Surgifoam, and Celox), and the solid volume fraction, surface area‐to‐volume ratio, and surface area per unit volume were determined (Figure S17A–C). Surgifoam presented the lowest solid volume fraction of these materials, followed by foam, composite, and Celox (Figure S17A), indicating that Surgifoam presented the highest quantity of void space and the least amount of solid material for a given volume. The inverse trend is present for surface area‐to‐volume ratio (Figure S17B). Celox had the lowest surface area‐to‐volume ratio, followed by composite, foam, and Surgifoam. Analysis of the surface area per unit volume, which considers the surface available for interaction with blood in a given volume of space (Figure S17C) revealed that Surgifoam presented the least surface per unit volume, followed by Celox, foam, and composite.

Because of the differences in surface area and volume fraction trends among these products (Figure S17D), and the lack of a direct correspondence with the observed in vivo clotting time (Figures 4, 5) and blood loss (Figures 4, 5), surface area alone is unlikely to be the sole determinant of hemostatic performance. The in vivo data demonstrates comparable hemostatic efficacy for the composite, Celox, and Surgifoam, despite their distinct structural and material properties. While it is plausible that a combination of surface availability and material–blood interactions contribute to the observed outcomes, the interplay between blood diffusion, surface accessibility, and hemostatic activity was not explicitly examined in this study. Accordingly, definitive conclusions regarding structure–function relationships cannot be drawn at this stage, and further targeted studies will be required to elucidate the mechanisms underlying material‐dependent hemostatic behavior.

In addition to the demonstrated in vivo hemostatic efficacy, the composite's ease of delivery and favorable storage characteristics suggest potential utility in civilian and military settings. Although the composite showed accelerated degradability in vitro, its in vivo degradation was not directly assessed. Overall, space‐filling expansion, rapid blood uptake, interactions with blood cells and platelets, and procoagulant activity are likely contributors to the observed hemostatic performance in vivo, warranting further investigation.

### 2.6 Expandable Composite Demonstrates Biocompatibility in a Rat Subcutaneous Implantation Model

**FIGURE 6:**
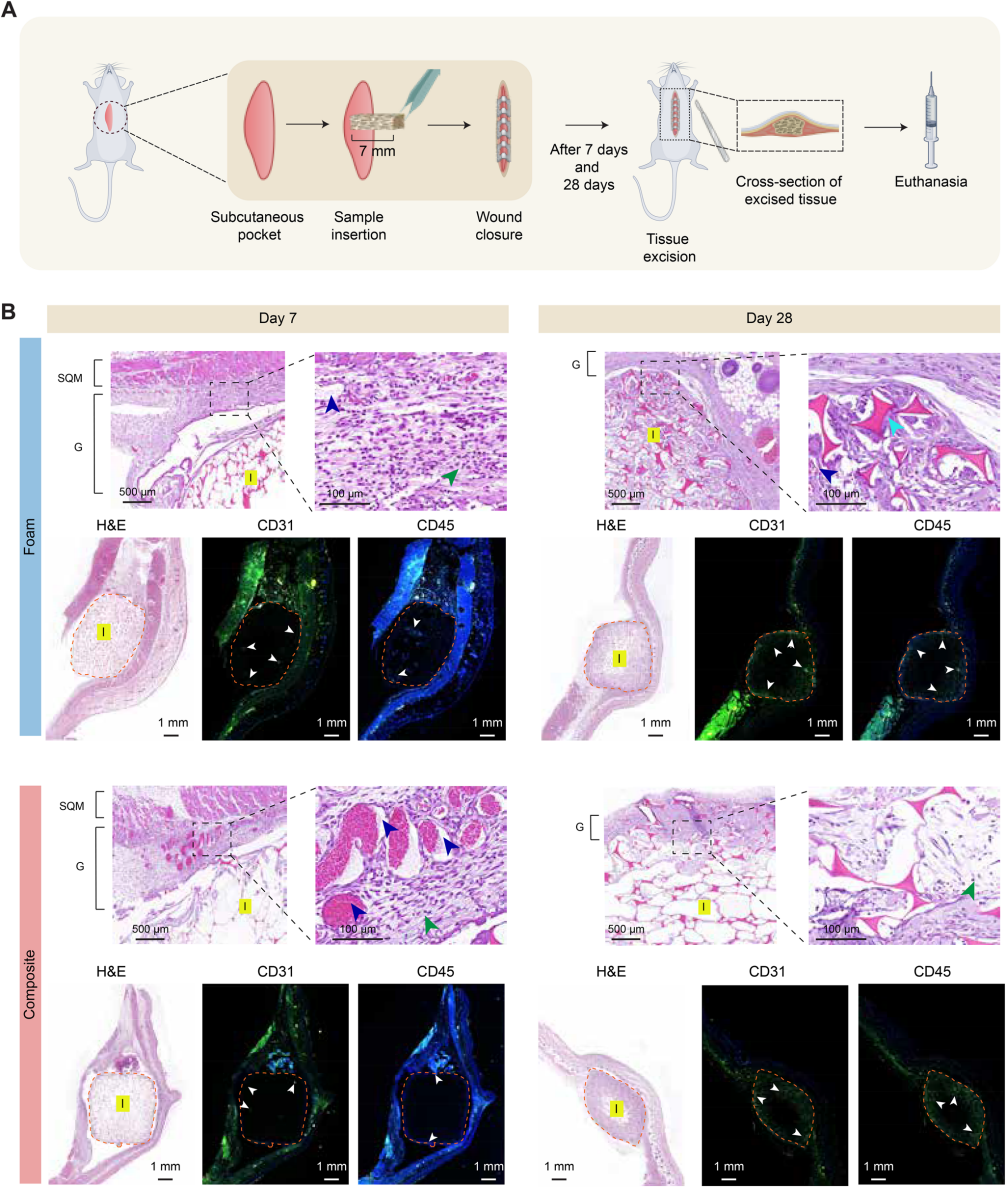
Subcutaneous implantation model to evaluate in vivo biocompatibility of expandable composite hemostat. (A) Surgery schematic of rat subcutaneous implantation model. The schematic was created partially using Biorender.com. (B) H&E images of tissue excised around the sample at the implantation site and immunofluorescence (IFC) images staining for CD31 and CD45. SQM: subcutaneous muscle; G: granulation tissue, I: implant fragments. The blue arrow indicates blood vessels, and the green arrow indicates lymphocytes. Orange dashed lines indicate the implant border within the subcutaneous pocket. White arrows indicate staining within the implant border. Image brightness was uniformly enhanced using Adobe Photoshop. Original images and modified images are available in Zenodo repository.

Finally, the composite's biocompatibility was evaluated in vivo using a rat subcutaneous implantation model. Sterilized foam, composite, and Surgifoam samples were surgically implanted in a subcutaneous pocket of Sprague Dawley rats and remained implanted for 7 and 28 days (Figure 6A). After this period, the samples and surrounding tissues were excised, fixed, and stained to observe the cellular morphology around the implantation site to assess necrosis and inflammation. Both the foam and composite implant sample fragments were able to be identified following tissue excision, but the Surgifoam samples appeared to have been completely degraded and could not be identified. This result can be attributed to the “melting” characteristic of Surgifoam, which was seen during in vitro blood clotting studies as well as to the extended degradation timeline of the foams and composites. Over an extended period, the foam and composites are expected to be disintegrated and degraded with enhanced cellular infiltration [24]. The H&E staining after 7 days confirms the presence of a vast number of clear healthy tissues with muscle fibers and blood vessels that have been observed around both foam and composite sample fragments, indicating the potential of neovascularization within the implant over a longer period. This result is further supported by CD31 staining around the implant site after 7 days (Figure 6B). However, a few leukocytes have also been found around the implanted samples in both H&E staining and CD45 staining, demonstrating an inflammatory response to a certain extent, which is consistent with a typical early wound‐healing response (Figure 6B) [34].

Upon analyzing the histological score, both the foam and composite displayed mild‐moderate inflammation. Additionally, both samples demonstrated only minimal or no necrosis, and no significant change in cellular morphology was observed in any of the major organs, including lungs, kidney, liver, and spleen for both foam and composite, indicating no systemic toxicity (Figure S18 and S19). After 28 days of implantation, macrophages and leukocytes were observed with blood vessels around the implant in both foam and composite, where both the samples demonstrated no necrosis. Both CD31 and CD45 staining appear to be increased in both foam and composite samples, especially around the borders of the implants (Figure 6B). The inflammatory reaction was noted to be higher, compared to day 7 (Figure S18), which is expected to be resolved over a longer time duration [22]. Based on previous studies on similar shape‐memory foam, we do not expect to observe long‐term extensive immune response. Rodriguez et al. evaluated biocompatibility and immune response of polyurethane‐based shape‐memory foam in a porcine aneurysm model over a span of 90 days, where after 1‐month, partial healing was confirmed after histological evaluation, and minimal inflammatory response was observed after 3 months post‐implantation. 5%–10% inflammatory cell infiltrates, including macrophage, neutrophil, and lymphocytes were observed after a month, showing mild inflammation, whereas the number of inflammatory cell infiltrates were reduced to less than 5% after 3 months with mild inflammation score, with increased neovascularization. Further, connective tissue substrate with minimal inflammatory cells was observed within the scaffold for 3 months, which confirmed the presence of granulation tissue, making the foam biocompatible with effective host‐response [66, 74]. In similar studies, low‐density polyurethane foam illustrated biocompatibility in larger aortic aneurysm models and canine models [21, 75]. In another study, Herting et al. introduced polyurethane shape memory foam as a coating on coils as embolic device and assessed biocompatibility in a rabbit aneurysm model over 30, 90, and 180 days. More complete healing with polyurethane‐based shape memory foam coated group was observed with higher healing score after 90 days, compared to the control, where the same trend was present in collagen deposition at the site of implantation as well. Though a higher inflammation score was obtained after 30 days, the inflammatory response was mitigated after 90 days, indicating that the polyurethane foam is not expected to promote significant immune response in long term [22].

On the other hand, injectable hydrogel containing nanosilicate and gelatin has been studied for hemostatic efficacy and as an endovascular embolic agent. Avery et al. showed negligible myeloperoxidase staining around the vessel wall, which suggested confined inflammation in the lumen [59]. Moreover, predominant expression of CD68 and proliferating cell nuclear antigen (PCNA) staining highlighted clearance of residual hydrogel and active cellular proliferative activity, indicating cellular remodeling with presence of connective tissue [59]. These results suggest a promising long‐term host response by the components of the composite present in this study. Though an initial mild‐moderate inflammatory response is expected, based on literature, the response will be mitigated in later time points, making the composite suitable to deliver at the bleeding site without extensive immune response. The primary aim of fabricating the composite was to develop an efficient expandable hemostat with an excellent procoagulant ability that can rapidly fill in the injury cavity to accelerate clotting and must be easy to be delivered both in battlefield and civilian settings to increase the “golden hour” of the patients, suffering from noncompressible hemorrhage, and enable the patients to receive definitive medical treatment. While obtaining care, the expandable hemostats are to be removed surgically within a short period [7]. The in vivo biocompatibility results suggest no severe inflammation, with minimal necrosis, supporting the composite hemostat's overall good biocompatibility, which makes it a suitable therapeutic alternative to combat noncompressible hemorrhage rapidly.

### 2.7 Limitations

Several limitations were present in this study, which must be addressed prior to implementation in humans. Although multiple human blood donors were included in the in vitro studies, all donors were known to be healthy and did not possess any coagulation deficiencies. Additional exploration into the hemostatic ability of this composite material in the presence of clotting disorders is needed. Owing to the dip coating method, it is very difficult to standardize the exact amount of Gentamicin present in each sample when Gentamicin is loaded in the hydrogel to fabricate the composite. The in vivo study with a biopsy punch injury also presented notable challenges regarding the evaluation of the hemostatic composite. The biopsy punch was made using a 6‐mm diameter punch, and the inserted samples were 5‐mm cubes, which resulted in one of the six samples being inserted directly into the biopsy punch cavity, and the remaining five samples being positioned on the surface of the liver and not in direct contact with the injury. The decreased contact between the samples and the bleeding tissues in the biopsy punch model may have contributed to the observed lower significance between the foam and composite structures relative to the laceration model. In future experiments, a larger biopsy cavity in a porcine model will be carried out, which may be beneficial to place multiple samples in direct contact with the damaged tissues. In addition, the amount of a sample selected for testing was set to be comparable to the composite structure; that is, the foam, Surgifoam, and composites were all administered as six individual cubes, while the Celox was standardized as 50 mg, which was an equivalent weight to 6 composite cubes. Due to this scheme, the mass of samples of foam and Surgifoam was less than that of the composites or Celox, which may have impacted results. This scheme was also used during in vitro studies (i.e., equivalent volumes of PBS, 0.1% Triton‐X, composite cubes, and equivalent masses of composite cubes and kaolin powder). We hypothesize that due to rapid expansion to absorb plasma and adhering blood cells, delivery of multiple samples at the biopsy punch site will lead to a reduced clotting time and amount of blood loss, statistically significant between foam and composite. The reader should also note that the photographic images taken for the in vivo study (Figure 4B and 5B) were not taken from animals that were also used for data analysis (i.e., not included in Figure 4C,D or Figure 5C,D), due to the extended time required for obtaining clear images. The surgical methods were the same as those for the analyzed animals. The histology images shown in these figures were taken from animals that were used for data collection. The histology scores displayed in Figures 4E and 5E were obtained by subjective analysis from an expert pathologist. It is possible that scores obtained from another pathologist may vary. The pathologist was the same for all scores to mitigate any potential variation between analyses. Surgifoam, being a gelatin‐based material, shrinks rapidly upon contact with blood. Consistent with the “melting characteristics” of the Surgifoam, confirmed by several in vitro analyses, the Surgifoam seemed to be disintegrated and degraded quickly upon in vivo implantation, which made it extremely challenging to identify Surgifoam after 7 days and analyze the subcutaneous implantation results accordingly.

## 3 Conclusions

In this study, we developed an expandable nanocomposite hemostat with rapid expansion, superior cytocompatibility, biocompatibility, and accelerated clotting efficiency. The material's shape memory polymer foam enables rapid fluid absorption, while its nanocomposite enhances adhesion of red blood cells and platelets, and activates the intrinsic and extrinsic pathways of blood coagulation, leading to effective hemorrhage control. Upon delivery of the composite, the in vivo animal study showed significantly reduced clotting time and blood loss, with minimal tissue damage. Further, in vivo subcutaneous implantation of the composite confirmed its biocompatibility, making it suitable to deliver at the site of injury. The integrative strategy of developing the composite leverages the synergistic effects of each component to accelerate the overall hemostasis process. This hemostat offers remarkable transformative potential for managing noncompressible hemorrhage, enabling faster recovery and reduced mortality in both military and civilian trauma scenarios.

## 4 Experimental Section

### 4.1 Foam Synthesis and Validation

The hemostatic composites described in this study were fabricated by combining a shape memory polymer foam with a diluted version of a hemostatic hydrogel. The shape‐memory foam composed of N,N,N′,N′‐tetrakis(2‐hydroxypropyl) ethylenediamine (HPED; Sigma Aldrich, USA), triethanolamine (TEA; 98% Alfa Aesar Inc., USA), and hexamethylene diisocyanate (HDI; TCI Chemicals, USA) was synthesized according to previously published protocols [36, 76]. This foam is fabricated with a composition to satisfy the isocyanate equivalents with HPED (24%), TEA (35%), and water‐driven urea linkages (41%). Samples were taken from the top, middle, and bottom portions for characterization. To evaluate the density of the foam, 3 rectangular sections were taken from each portion, measured, and weighed. A thin slice was taken from each portion to evaluate the porosity using a VHX stereomicroscope (Zeiss, Germany) and a differential scanning calorimeter (TA Instruments, USA) was used to identify the glass transition temperature. Following characterization, the bulk foam was then sectioned into 5‐mm cubes using a heated wire and compressed as described below.

### 4.2 Nanocomposite Synthesis

A nanocomposite solution was prepared from gelatin (300 Bloom, Type A; Sigma Aldrich, USA), nanosilicates (Laponite XLG; BYK, USA), and deionized water. Various concentrations were produced to determine the optimal percentages of gelatin and nanosilicates (Figures S1–S4). The solution was stored at 4°C until further use.

### 4.3 Composite Fabrication

To prepare the hemostatic composites, the nanocomposite was reheated to approximately 40°C. The 5‐mm foam cubes were submerged in the nanocomposite and allowed to soak in an oven set to 60°C for 1 h to allow infiltration throughout the foam. The samples were then allowed to sit under vacuum at room temperature for 2 h to further facilitate infiltration within the foam. Cubes were then stored at ‐80°C overnight and then lyophilized (Labconco FreeZone, USA) overnight.

### 4.4 Sample Compression

Prepared samples were heated in an oven at 80°C for 1 h. This temperature was selected for being notably above the glass transition temperature (∼55–60°C) of the foam. The samples were then placed between the platens of a hot press (Carver, USA), which were set to 80°C and allowed to incubate for 5 min to ensure even heating throughout the sample. The samples were compressed to a height of approximately 1 mm using aluminum spacers as a guide. After 15 min of compression, the heating element of the platens was deactivated, and the samples were allowed to cool before removing the platens.

### 4.5 Physicochemical Characterization

Rheological characterization, including shear rate sweep and stress sweep of nanocomposite samples, was performed with a stress‐controlled rheometer (DHR‐2 Discovery hybrid rheometer, TA Instruments, USA). For each experiment, we utilized a 20‐mm parallel plate geometry and a gap height of 0.2 mm. A solvent trap was also used to prevent drying of the nanocomposite while the test was occurring. Foams and composite samples were analyzed via attenuated total reflectance‐Fourier transform infrared (ATR‐FTIR) spectroscopy (ALPHA Infrared Spectrometer with a diamond ATR crystal, Bruker, USA) to determine the presence of any chemical changes due to hydrogel infusion and UV exposure. For all in vitro cell studies and in vivo studies, compressed foam and composites were sterilized under UV (365 nm) for a total of 1 hr split between both sides. Peaks were observed and compared to those seen in previously published literature for the polyurethane foam and for nanosilicates [36, 77, 78, 79]. Samples were also evaluated via scanning electron microscopy (SEM). Samples were prepared as described above, sectioned into thin slices, and sputter coated with platinum‐palladium (Sputter Coater 208 HR, Cressington, UK). The surface topography and structure of each sample were observed using a 5 kV electron beam (JSM‐7500F, JEOL, Japan; Research Resource ID: SCR_022202). Energy dispersive X‐ray spectroscopy (EDS) was used to identify the presence of magnesium and silicon in the composites. Finally, samples were imaged using micro‐computed tomography (Skyscan 1272 XRM, Bruker, USA) to examine the complex internal structure. CT scans were reconstructed using the CTvol program (Bruker, USA) and analyzed using the CTan program (Bruker, USA) on three non‐overlapping volumes of interest.

### 4.6 Expansion

The ability of the compressed composite and foam samples to expand and recover their primary shape was evaluated by observing the volume change of the samples when added to a bath of 1X PBS at approximately 37°C. Samples were compressed as described above. PBS was preheated to approximately 37°C in an oven and the temperature was confirmed with a digital thermometer. The samples were then dropped into the PBS bath and their expansion was video‐recorded from the side. Frames from the video were selected at specific time points (every 5 s for the first min, 75, 80, 85, 90, and every 15 s thereafter until 3 min). The height of the sample at each of these time points was measured using ImageJ (NIH) and normalized to the initial height of the sample at time t = 0 s.

### 4.7 Expansion Force

Expansion force measurement was conducted in a physiological environment according to our previously published protocol [51]. Briefly, an immersion chamber held PBS at physiological temperature and a forced‐convection oven maintained the temperature throughout the test. A blank test was conducted with no sample to determine the buoyancy force due to the presence of the PBS. This force was subtracted from the raw force values determined for each sample to calculate the force due to axial expansion.

### 4.8 Swelling Percentage

The swelling percentage was measured using a previously established protocol with minimal modification [80]. Briefly, after measuring the initial weights, samples were submerged in 1X PBS (pH 7.4). Then, the well plate was incubated at 37°C for 30s, 1 min, 3 min, 5 min, 10 min, 15 min, and 30 min. Following incubation after each time point, each sample was taken out from the well plate and excess water was removed accordingly. Then, the sample weight was recorded, and the swelling percentage was calculated using Equation (1). Each experimental group was repeated three times (*N* = 3).

(1) $$\mathrm{Swelling}\;\mathrm{percentage}=\frac{\left(\mathrm{Ww}-\mathrm{Wd}\right)}{\mathrm{Wd}}\times 100\%$$

where, W_w_ ‐ sample mass after swelling

W_d_ ‐ initial sample mass

### 4.9 Degradation

Three separate degradation studies, including hydrolytic degradation, oxidative degradation, and enzymatic degradation were performed. For the hydrolytic degradation, after recording the initial weights, one uncompressed foam and composite of 5 mm dimension was submerged separately in 5 mL of 0.1 N NaOH (Research Products International, USA) solution with a volume ratio of 1:40 (sample: NaOH solution) in glass vials. The samples were incubated at 37°C for 1, 3, 5, 10, 20, 30, 40, and 50 Days. After every three days, the solutions were replaced with fresh 5 mL aliquots of 0.1 N NaOH to keep the pH and ion concentration constant. After every time point, following rinsing with DI water, samples were stored at −80°C overnight in the freezer. Following freezing, samples were lyophilized for 48 h in the lyophilizer and the mass was recorded. The degradation rate was calculated using Equation (2). The oxidative degradation was performed using the same protocol, mentioned above, except for using 20% H_2_O_2_ (Sigma‐Aldrich, USA) solution. For the enzymatic degradation, 1 mg/mL collagenase type IV (Worthington, USA) solution was used for the study, and the samples were incubated at 37°C for 1, 3, 5, and every 5 days thereafter until 30 days. The rest of the protocol was the same as the abovementioned hydrolytic degradation study. In vitro degradation was also assessed with 0.1 M acetate buffer (pH 4.0), phosphate buffer saline (pH 7.4), and 0.1 M carbonate buffer (pH 9.0), 10% H_2_O_2_, and 30% H_2_O_2_ solutions. The samples used in 20% H_2_O_2_ solution for Figure 2G and Figure S7E are different and carried out as independent studies. For all the degradation studies, the remaining mass of the samples was calculated using Equation (2).

(2) $$\mathrm{Mass}\;\mathrm{remaining}=(1-\frac{\left(\mathrm{Wi}-\mathrm{Wf}\right)}{\mathrm{Wi}})\times 100\%$$

Where, W_i_ – the initial mass of the sample

W_f_ – the final mass of the sample

### 4.10 In Vitro Studies

#### 4.10.1 Human Blood

Citrated human blood was obtained ethically from donors with informed consent under IRB 2022‐0501D. All procedures regarding human blood followed protocols approved by the Institutional Review Board at Texas A&M University. Experiments involving human blood were conducted within a biosafety cabinet in accordance with Institutional Biosafety Committee requirements. All experiments involving human blood were performed with three biological replicates from three healthy individual donors, with no conflict of interest.

#### 4.10.2 Bovine Blood

Bovine blood was purchased from Texas A&M University Veterinary Medicine. Blood was collected into commercial blood collection bags and anticoagulated with CPDA‐1 (citrate phosphate dextrose adenine, variation 1) anticoagulant. Blood was stored at 4°C and used within 2 weeks of the collection date.

#### 4.10.3 Whole Blood Clotting

The whole blood clotting test was performed by the test tube inversion method, using a modified previously established protocol [4]. Briefly, fresh anticoagulated whole human blood and compressed foam and composites were kept in the incubator at 37°C for 10 min before use. Anticoagulated blood was recalcified using 0.1 M CaCl_2_ (VWR Life Sciences, USA) with a 9:1 volume ratio. 3 compressed foams, composites, and Surgifoam (Ethicon, USA) of 5 mm dimension were put in a separate polystyrene tube and 1 mL of recalcified blood was added. 25 mg of kaolin (Sigma Aldrich, USA) was added to the tube as it equals the weight of 3 pieces of composite. Next, each tube was inverted at 15 s intervals to check for blood flow and clotting was identified when no visible blood flow was present. Surgifoam and Kaolin were considered positive controls and no treatment group was considered negative control. Each experimental group was repeated four times (*N* = 4). Whole blood clotting with bovine blood was also performed using the same protocol.

#### 4.10.4 PT and APTT Assay

The effect of the samples on blood coagulation pathways was assessed using Activated partial thromboplastin time (APTT) and prothrombin time (PT) assays. Citrated whole blood was centrifuged at 1500 × g for 15 min. The supernatant was collected as platelet‐poor plasma (PPP). All reagents, samples with 5 mm dimensions, and PPP were incubated at 37°C for 3 min. For the APTT assay, APTT reagent (Thermo Scientific, USA) and 0.025 M CaCl_2_ were added to the PPP in a 1:1:1 ratio (V/V) and the APTT was recorded using a stopwatch. For the PT assay, the PT reagent (Thermo Scientific, USA) to PPP ratio was maintained at 2:1 (V/V) to incubate the samples, and the PT was recorded using a stopwatch. [4, 81, 82].

#### 4.10.5 Blood Clotting Index

The blood clotting index (BCI) was measured using a previously established protocol with minimal modification [83]. Briefly, fresh anticoagulated whole human blood and compressed foam and composites were kept in the incubator at 37°C for 10 min before use. Anticoagulated human blood was recalcified using 0.1 M CaCl_2_ with a 9:1 volume ratio. Next, each sample was placed into a well plate and 100 µL of recalcified bovine blood was added to the surface of each sample. The samples were incubated at 37°C and 40 rpm for 1, 3, 5, and 10 min. After each time interval, 2 mL of DI water was added carefully along the wall of the well plate to avoid rupturing the clot and subsequently incubated at 37°C and 40 rpm for 10 min to rupture the unbound red blood cells. The supernatant was collected and the optical density (OD) was measured at 540 nm with a plate reader (Tecan Group Ltd, Tecan Infinite 200Pro M Plex, Switzerland). Surgifoam and no treatment were considered as positive and negative controls, respectively. The OD value of the negative control was taken as a reference value to calculate the BCI, using the following formula mentioned in Equation (3). Each experimental group was repeated three times (*N* = 3). Representative images were captured with all experimental groups. The same experiment was performed using whole bovine blood as well.

(3) $$\mathrm{BCI}=\frac{\mathrm{OD}\;\mathrm{sample}}{\mathrm{OD}\;\mathrm{reference}}\times 100\%$$

#### 4.10.6 Expansion in Plasma

The expansion ability of the compressed samples was again evaluated in citrated human plasma in place of PBS to better approximate a physiologic condition. Expansion was monitored in plasma rather than whole blood due to the need to observe the sample's expansion and the opaque nature of whole blood. To obtain plasma, the supernatant was collected after centrifuging citrated whole blood for 5 min at 1700 g. Plasma was warmed to approximately 37°C prior to use and kept warm during the experiment using an infrared heating lamp. The analysis procedure follows as described in the “Expansion” methodology.

#### 4.10.7 Clot Formation and Platelet Activation Observation

SEM was used to observe blood clots and aggregation of platelets within the samples. For the observation of blood clots, samples were allowed to incubate in warmed, recalcified human blood (9:1 blood: calcium chloride). Samples were then fixed for 48 h in 4% paraformaldehyde (Fischer Scientific, USA) in PBS at 4°C. Samples were washed with PBS to remove paraformaldehyde and subsequently subjected to a graded ethanol dehydration series (20%, 30%, 40%, 50%, 60%, 70%, 80%, 90%, 100%, 100%). Finally, samples were immersed in hexamethylene disilazane (EMS Chemicals, USA, 999‐97‐3) and allowed to dry for at least 4 h. Samples were then mounted, sputter coated and imaged as previously described.

For the observation of platelets, a platelet solution was extracted from whole blood. Platelets were isolated according to Abcam protocol for “Isolation of human platelets from whole blood” [84]. Samples were then incubated in platelet solution and prepared following the same procedure as the whole blood samples above.

#### 4.10.8 Red Blood Cell Adhesion

A 5% red blood cell solution was prepared from whole blood by repeatedly centrifuging at 1700 g for 5 min and washing the pellet with PBS. Samples were placed into microcentrifuge tubes and incubated in the red blood cell solution for 1 h at 37°C. After 1 h, the samples were washed with PBS. All of the solution was aspirated. Each sample was then treated with 1% Triton‐X 100 in deionized water and vortexed for 10 s to release all cells from the sample and incubated for another hour at 37°C. Afterward, the samples were centrifuged at 2000 g for 5 min and the absorbance of the supernatant was read at 540 nm using a plate reader (Agilent, BioTek Cytation 5 Cell Imaging Multimode Reader, USA). A standard curve of varying concentrations of red blood cells was used to quantify the cells adhered to the sample. Surgifoam acted as a positive control; blank tubes served as a negative control.

#### 4.10.9 Platelet Adhesion

The adhesion of platelets to the hemostatic material was evaluated in vitro. A solution of platelets was prepared following Abcam's Isolation of Human Platelets protocol. Samples were placed into microcentrifuge tubes and incubated with the platelet solution for 1 h at 37°C. After 1 h, the samples were washed three times with 0.5 mL of PBS. All of the solution was aspirated. Each sample was then treated with 0.5 mL of 1% Triton‐X 100 in deionized water. The samples were then vortexed for 10 s to release all cells from the sample and incubated again for 1 h at 37°C. The supernatant was sampled into the 96‐well plate and relative platelet adhesion was quantified using an LDH Assay kit (Cayman Chemical, USA). A sample of only the platelet solution and 1% Triton‐X 100 served as a “maximum release” sample to represent the lysis of all available platelets. Blank tubes (negative) and Surgifoam (positive) served as controls. Relative platelet adhesion was quantified using the equation (4):

(4) $$\begin{array}{ccc} & & \mathrm{Platelet}\mathrm{adhesion}\\ & & =\frac{\left(\mathrm{sample}\;\mathrm{absorbance}-\mathrm{neg}.\;\mathrm{control}\;\mathrm{absorbance}\right)}{\left(\mathrm{maximum}\;\mathrm{release}\;\mathrm{absorbance}-\mathrm{neg}.\;\mathrm{control}\;\mathrm{absorbance}\right)}\\ & & \;\times 100\% \end{array}$$

#### 4.10.10 Blood Cell Infiltration

Foam, composite, and Surgifoam samples (*N* = 3) were incubated with fresh, citrated whole human blood for 30 min at 37°C. Samples were then removed from the blood and fixed in 4% paraformaldehyde at 4°C for at least 72 h. Fixed samples were cryopreserved in optimal cutting temperature media overnight. Embedded samples were cryosectioned (Cryostat Leica CM1950, Leica Biosystems, Germany) at 80 µm thickness to produce firm sections with sample material present. Sections were then imaged under 4X and 8X magnification using a Zeiss Discovery V8 SteREO microscope (Zeiss, Germany). Images were adjusted for color in Adobe Photoshop (Adobe, USA). Original images and Photoshop files are available in Zenodo repository.

#### 4.10.11 Flow Cytometry

The platelet activation ability of nanosilicates was evaluated through flow cytometry. A solution of platelets was prepared following Abcam's Isolation of Human Platelets protocol. 100 µL platelet solution was taken in a microcentrifuge tube and incubated with increasing concentrations of nanosilicates (10, 25, and 50 µg/mL) for 1 h at room temperature. Following treatment, the platelet solution was centrifuged at 750 g for 10 min and then washed twice with PBS. The platelets were fixed with 2% paraformaldehyde for 30 min at room temperature, followed by washing in PBS twice and resuspended in 100 µL FACS buffer (PBS+ 1% BSA). The fixed platelets were stained with CoraLite Plus 647 Anti‐Human CD41 (Proteintech, USA) and anti‐human CD62P‐FITC (Proteintech, USA) antibodies for 30 min in dark at room temperature. The stained platelets were washed with FACS buffer, resuspended in 500 µL of the same buffer, and analyzed using MACSQuant Analyzer 16 (Miltenyi Biotec, Germany). Non‐treated platelets and platelets incubated with 0.2 U/mL Thrombin (Cytiva, USA) for 10 min were used as negative and positive controls, respectively. For studying phosphatidylserine externalization, Annexin V‐FITC (APExBIO, USA) staining was performed as per the manufacturer's protocol.

#### 4.10.12 Hemolysis

Hemocompatibility of the samples was observed via hemolysis. A 4% red blood cell solution was prepared from whole blood by repeatedly centrifuging at 1700 g for 5 min and washing the pellet with PBS. Samples were placed into microcentrifuge tubes and incubated in the red blood cell solution for 1 h at 37°C. After 1 h, the samples were centrifuged, and the absorbance of the supernatant was read at 540 nm using a plate reader (Agilent, BioTek Cytation 5 Cell Imaging Multimode Reader, USA). Surgifoam acted as a clinical control; 0.1% Triton‐X 100 served as a positive control, and PBS served as a negative control. Hemolysis was calculated using the equation (5).

(5) $$\begin{array}{c} \mathrm{Hemolysis}=\frac{\left(\mathrm{sample}\;\mathrm{absorbance}-\mathrm{neg}.\;\;\mathrm{control}\;\mathrm{absorbance}\right)}{\left(\mathrm{pos}.\;\;\mathrm{control}\;\mathrm{absorbance}-\mathrm{neg}.\;\;\mathrm{control}\;\mathrm{absorbance}\right)} \end{array}\times 100\%$$

#### 4.10.13 Cell Culture

NIH 3T3 mouse fibroblast (CRL‐1658, ATCC, USA) was cultured with Dulbecco‐modified eagle medium (DMEM) high glucose (Cytiva‐Hyclone, USA) and 1% penicillin/streptomycin (100U/100 µg/mL; Thermo Fisher Scientific, USA) under an aseptic condition at 37°C with 5% CO_2_ with media refreshed every two days. When 80% confluency was reached, cells were passaged using 0.5% trypsin‐EDTA (Thermo Fisher Scientific, USA) at approximately 7500 cells per cm^2^ for expansion.

#### 4.10.14 Cytocompatibility

The cytocompatibility was performed using the Alamar Blue and the Live/Dead assay with an indirect transwell method. Cells were seeded on a 24‐well plate at a density of 5000 cells/well, and sterilized foam and composite were placed in the transwell. The media was added to submerge the samples completely, and cytocompatibility was measured based on the leachables released from the samples. The 24‐well plate was kept in the incubator at 37°C with 5% CO_2_. The Alamar Blue assay (Biorad, USA) was performed on days 1, 3, 5, 7, 10, and 14 with the tissue culture plate (TCP) acting as a positive control. Readings were taken at 570 nm and 600 nm with a plate reader (Tecan Group Ltd, Tecan Infinite 200Pro M Plex, Switzerland). Percent reduction was calculated and viability was calculated by normalizing percent reduction value after each day with percent reduction value after day 1. A live/dead assay was performed on days 1, 3, 5, 7, 10, and 14 using Calcein AM (Biotium, USA) and Ethidium Homodimer II (Biotium, USA) with the tissue culture plate (TCP) acting as a positive control. Images were captured with a fluorescence microscope (AX10, Zeiss, Germany).

#### 4.10.15 Antibacterial Study Cell Culture

Luria Bertani (LB) broth (BD Difco, USA) was prepared with dH_2_O, sterilized, and stored at room temperature. LB‐agar plates were prepared with deionized water and 1.5% agar. *E. coli* (DH5α) cultures were grown in LB broth [85]. Overnight cultures of *E. coli* were prepared and the OD of the cultures were adjusted by following our previously published methods [86]. An OD of 1.25 at 600 nm was obtained for all replicates and the solution was diluted further to confirm the viable cell count. The solution was then plated on LB‐agar plates and incubated at 37°C for 24 h and the CFUs were counted. The bacterial suspensions had ∼ 1 × 10^9^ CFU mL^−1^, which was diluted to obtain a final concentration of ∼ 1 × 10^6^ cells mL^−1^.

#### 4.10.16 Antibacterial Testing

Both sterilized compressed foam and composites and foam and composites infused with 2 µL of Gentamicin (Quality Biological, USA) were used for this study. The samples were added to 1 mL of LB broth in a centrifuge tube and then 10 µL (∼ 1 × 10^4^ cells) of the diluted bacterial solution was added. These tubes were then incubated at 37°C at 200 rpm. After 24 h, an aliquot from the tubes was diluted (as necessary) and plated on LB‐agar plates before incubating 37°C for 24 h and the CFUs were counted. Gentamicin was used as a positive control. For Gentamicin‐loaded samples, an aliquot of 100 µl was plated (zero dilution) on LB‐agar plates. For non‐gentamicin‐loaded samples, diluted aliquot was plated on LB‐agar plates. Representative images of LB‐agar plates showing *E. coli* colonies when 10^4^ CFU/mL of *E. coli* was exposed to different samples after 24 h.

In another approach, Gentamicin was loaded in the hydrogel in 1:10 ratio to fabricate the composite. Gentamicin was added to the DI water in 1: 10 volume ratio, and foams were incubated for 24 h before use. Then, gentamicin‐loaded composites and foams were used to evaluate the antibacterial efficacy of the sample for different time intervals. The samples were added to LB broth with 10 µL (∼ 1 × 10^4^ cells) of the diluted bacterial solution and incubated at 37°C and 200 rpm. Every 24 h, the samples were removed, washed, and placed into fresh LB broth with diluted bacterial solution (∼ 1 × 10^4^ cells). Every 24 h, before the samples were removed, an aliquot from the culture was taken, diluted, and plated on LB‐agar plates. The CFUs were counted to denote the antibacterial properties of foams and composites. After each time point, the same sample was incubated in fresh media for 24 h and aliquots were taken to culture the bacterial cells to check the release of Gentamicin from each sample. Spot test was also performed to take representative images after every time point.

### 4.11 In Vivo Studies

All experiments involving animal procedures were conducted according to the animal use protocol (IACUC2022‐0010D/2024‐0301 for liver laceration and liver biopsy punch; IACUC2023‐0061 for subcutaneous implantation) approved by the Texas A&M University Institutional Animal Care and Use Committee. All animal surgeries were performed on Sprague Dawley (SD) rats (3–6 months old, weighing between 200 and 500 g) obtained from Envigo, Indianapolis, USA. Four rats (2 M, 2F, *N* = 4) were randomly selected and treated with the same experimental groups for all animal studies. For all animal surgeries, animals were chosen randomly and anesthetized by an intraperitoneal cocktail injection of ketamine and xylazine. Once, under anesthesia, animals were kept on a heated platform at 37°C to maintain homeostasis, and artificial gel (Aventix Animal Health, Canada) was applied to animal eyes to avoid dryness. For the liver laceration and liver biopsy punch model, the hair on the abdomen was trimmed and the incision area was disinfected using 70% ethanol and 4% Chlorhexidine gluconate (MP Biomedicals). For all in vivo studies, Surgifoam and Celox serve as positive controls, and no treatment serves as negative control.

#### 4.11.1 Rat Liver Laceration Model

The previously established protocol was modified and used accordingly for this study [34]. The abdominal cavity was opened using surgical scissors up to ∼1 inch below the ribcage of the animals, and the abdominal cavity was exposed using clamps. Then, the serous and body fluids were carefully removed around the liver using surgical nonwoven gauze (Medline Industries, USA). Next, pre‐weighed non‐absorbent surgical gauze was placed, and ventral laparotomy was performed to expose the liver. The left medial liver lobe was injured by making a laceration of ∼1.5 cm using a surgical scalpel 10 blade (Swann Morton, UK). Following the laceration, six pieces of foam with 5 mm dimensions were applied at the laceration site. Same number of samples were applied with other experimental groups as well. For the Celox, 50 mg of the powder was applied, which equals the weight of six composites of 5 mm dimension. After applying the samples, bleeding was monitored at every 10 s intervals to ensure clotting and the time taken from the time of laceration to complete arrest of bleeding was recorded using a stopwatch and considered as clotting time. Thereafter, the non‐absorbent surgical gauze was taken out and weighed to measure the amount of blood loss using Equation (6). Then the wound was closed using a wound clip to provide adequate closure and the animal was kept alive for 1 h under constant anesthesia. Post 1 h, the rat was euthanized with sodium pentobarbital (150 mg/kg). Next, the lacerated liver lobe was immediately excised and stored in 10% NBF (VWR Life Sciences, USA) with a volume ratio of 1:10 for histological analysis. During the surgical procedure, representative photographs were captured for each experimental group.

(6) $$\mathrm{Amount}\;\mathrm{of}\;\mathrm{blood}\;\mathrm{loss}=m_{1}-m_{0}$$

where, m_1_ ‐ Weight of non‐absorbent surgical gauze after clotting

m_0_ ‐ Initial weight of non‐absorbent surgical gauze before laceration

#### 4.11.2 Rat Liver Biopsy Punch Model

As mentioned in the liver laceration model, the previously established protocol was modified and used accordingly for this study [87]. A circular perforation wound was created on the left medial liver lobe, using a 6 mm biopsy punch (Medline Industries, USA). Following the biopsy punch, 6 pieces of foam with 5 mm dimensions were applied at the biopsy punch site. The same number of samples were applied for other experimental groups as well. Compressed foam and composite were applied using a 3D‐printed syringe, printed using polylactic acid filament (PLA) on a Creality Ender 3‐Pro 3D printer (Creality, China). For Celox, 50 mg of Celox was applied, which equals the weight of 6 pieces of the composite of 5 mm dimension. The rest of the procedure was followed as described in the liver laceration model.

#### 4.11.3 *Ex Vivo* Porcine Liver Injection

A porcine liver was obtained through the tissue share program at Texas A&M University. The liver was held under a heat lamp to maintain the temperature at approximately 37°C. A 6‐mm biopsy punch cavity was made in the surface of the liver tissue and bleeding was observed from the tissue. Composite samples were injected into this cavity using a 1 mL syringe with the end cut off. The samples were allowed to expand within the cavity and then removed for observation of the expansion.

#### 4.11.4 Subcutaneous Implantation

In vivo biocompatibility and host response were assessed using a subcutaneous implantation model on rats. For this study, samples with 7 mm dimension, including both foam and composite were applied and the compressed samples were sterilized under UV for 1 hour. Following anesthesia and disinfection, a ∼1 cm longitudinal incision was performed using sterile scissors on the back, and blunt separation was carried out to create the subcutaneous pocket [34]. Then, one compressed sample from each experimental group was surgically implanted in the pocket and the incision was clipped. Post‐surgery, rats were returned to their cages without limiting food and water intake. After 7 and 28 days, rats were euthanized, and tissues around the site of implantation, major organs, including lung, spleen, kidney, liver were surgically excised and stored for histological analysis.

#### 4.11.5 Histological Analysis

Animal liver tissue and subcutaneous tissue, and major organs were removed from euthanized animals and placed into 10% NBF for at least 24 h to ensure adequate fixation. Samples were maintained in NBF until all tissues were fixed. After fixation, liver tissues, and subcutaneous tissues were trimmed and embedded in paraffin wax. Tissues were sectioned at 5 µm and stained with hematoxylin and eosin using with standard protocol [88]. For liver tissues, the sections were analyzed for the severity of necrosis, inflammation, and fibrin deposition. Necrosis and inflammation were assessed as not present (0), mild (1), moderate (2), and severe (3). Fibrin accumulation was subjectively quantified as not present/ minimal amount present (0), mild amount present (1), moderate amount present (2), and abundant amount present (3). For subcutaneous tissues, the sections were analyzed for the severity of necrosis and inflammation. Necrosis and inflammation were assessed as not present (0), mild (1), moderate (2), and severe (3). The histology scores were determined for the tissue, localized to the site of liver injury, and around the site of subcutaneous implantation by a board‐certified veterinary pathologist, blinded to the treatment groups.

#### 4.11.6 Immunofluorescence Analysis

Samples were prepared following the above protocol for histological analysis. Unstained slides were stained with DAPI and additionally for either CD31 or CD45. CD31 was stained using an unconjugated rabbit host polyclonal antibody (Proteintech, USA) at a dilution of 1:500. CD45 was stained using an unconjugated rabbit host polyclonal antibody (Proteintech, USA) at a dilution of 1:500. The primary antibody was then stained with a CoraLite488‐conjugated Goat Anti‐Rabbit secondary antibody to reveal a green signal in the presence of the target with a dilution of 1:500.

#### 4.11.7 Slide Scanning

Stained slides for both histological and immunofluorescence analysis were imaged using Panoramic Scan II (3DHistech, Hungary) at 20X magnification to produce a series of mrxs files. Files were then converted to SVS format using SlideMaster (3DHistech, Hungary) to produce a single file without loss of quality or data.

#### 4.11.8 Statistical Analysis

Pre‐processing of data was limited to normalization or calculation of percentages and this pre‐processing is indicated where performed. No outliers or other data points were removed. Data is presented as mean with either standard deviation or standard error of the mean, as applicable, with the exception of the heat maps in Figures 4, 5, and Figure S18, which are presented as mean only. The information on data presentation is indicated in the figure caption. The sample size for each analysis is also indicated in the caption. Data analysis was conducted in Graphpad Prism 9. Categorical data was assessed using a one‐way ANOVA with Tukey's post hoc test unless otherwise specified. Levels of significance are indicated as: ^*^ *p* <0.05, ^**^ *p* <0.01, ^***^ *p* <0.001, ****p <0.0001.

## Author Contributions

S.B. and S.E.M. contributed to experimental design, figures, and data presentation, writing, proofreading, and editing. S.R., J.T., and S.F. contributed to experimental procedures, figures, and data presentation, proofreading, and editing. M. K. S – experimental design for the antibacterial study. S.M.G. contributed to the experimental design for the expansion force study. Y.J.H., S.J.H., and F.C. contributed to histopathology analysis and proofreading. T.H.W. contributed to supervision, proofreading, and editing. D.J.M. and A.K.G. contributed to conceptualization, funding, supervision, proofreading, and editing.

## Conflicts of Interest

The shape memory foam used in this study is licensed by Shape Memory Medical Inc., and the nanocomposite hydrogels are licensed by Boston Scientific. D.J.M. serves as the Director of Shape Memory Medical Inc. and holds equity in the form of shares and stock options. Neither Shape Memory Medical Inc. nor Boston Scientific provided funding or had any role in the design, execution, or interpretation of the research presented in this manuscript.

## Supporting information




**Supporting File 1**: advs73953‐sup‐0001‐SuppMat.docx.

**Supporting File 2**: advs73953‐sup‐0002‐VideoS1.mov.

## Data Availability

All raw data and analysis files are available at Zenodo DOI: 10.5281/zenodo.15297865 (Figures 1, 2, 3, 4, 5, Figures S10–S13) and Zenodo DOI: 10.5281/zenodo.17782422 (Figure 6, Figures S1–S9 and S14–S19).
