# Supplementary material for: Expandable Nanocomposite Shape‐Memory Hemostat for the Treatment of Noncompressible Hemorrhage
Source: Adv Sci (Weinh). 2026 Feb 6;13(23):e08439. doi: 10.1002/advs.202508439 (PMC13104121; doi:10.1002/advs.202508439)
Supplement: Supplementary file 1 — Supporting File 1: advs73953‐sup‐0001‐SuppMat.docx. [file ADVS-13-e08439-s001.docx]

**Supplementary Information**

**Expandable Nanocomposite Shape-Memory Hemostat for the Treatment of Noncompressible Hemorrhage**

*Saptarshi Biswas^1,#^, Sarah E. Miller^1, #^, Shounak Roy^1^, Jeevika Thazhaiseklvam^2^, Samantha Foster^3^, Manivannan Sivaperuman Kalairaj^1^, Sasha M. George^4^, Jones Yava-Hall^2,5^, Staci J. Horn^1^, Fred J. Clubb^2,5^, Taylor H. Ware^1,4^, Duncan J. Maitland^1^, and Akhilesh K. Gaharwar^1,3,4,6^**

^1^Department of Biomedical Engineering, College of Engineering, Texas A&M University, College Station, TX 77843, USA

^2^School of Veterinary Medicine and Biomedical Sciences, Texas A&M University, College Station, TX 77843, USA.

^3^Interdisciplinary Program in Genetics, Texas A&M University, College Station, TX 77843, USA

^4^Department of Material Science and Engineering, College of Engineering, Texas A&M University, College Station, TX 77843, USA.

^5^Department of Veterinary Pathobiology, Texas A&M University, College Station, TX 77843, USA.

^6^Center for Remote Health Technologies and Systems, Texas A&M University, College Station, TX 77843, USA.

^#^Equally contributed

*Corresponding author E-mail: gaharwar@tamu.edu (AKG); djmaitland@tamu.edu (DJM)


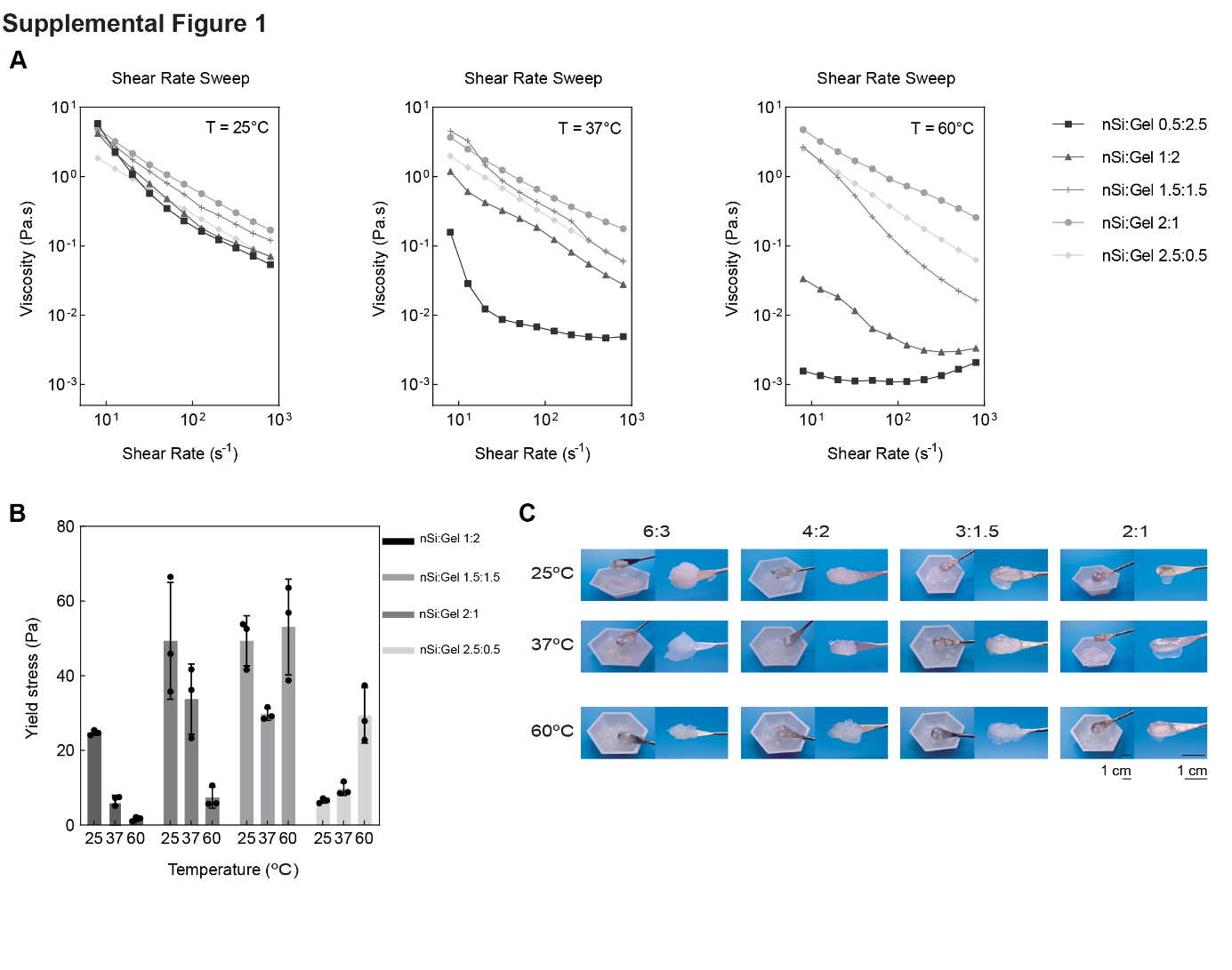


**Figure S1. Optimization of nanosilicate-gelatin hydrogel with varying concentrations.**

(A) Shear rate sweep analysis of different hydrogel concentrations at 25°C, 37°C, and at 60°C. Quantification of viscosity, N=3; data represented as mean ± standard deviation.

(B) Yield stress measurement of varying hydrogel concentrations at 25°C, 37°C, and at 60°C. N=3; data represented as mean ± standard deviation. Two-way ANOVA conducted in GraphPad Prism 9.

(C) Representative images of foams coated with varying concentrations of hydrogel at 25°C, 37°C, and at 60°C.

The viscosity of varying concentrations of hydrogel composition was evaluated. All hydrogel concentrations exhibited shear-thinning properties. The viscosity of all hydrogel concentrations was comparable at 25 °C, but at 37 °C, nSi-gel 0.5:2.5 showed less viscosity than the other hydrogel concentrations. Further, at 60°C, an evident decrease in viscosity was observed in nSi-gel 0.5:2.5 and nSi-gel 1:2, compared to other experimental groups. As gelatin is dissolved at 37°C, the hydrogel compositions with less nanosilicate and high gelatin wt% became less viscous, resulting in a less stable hydrogel system. Therefore, an optimized hydrogel composition demonstrating high-temperature stability was needed. A stress sweep was also performed for all hydrogel concentrations, and yield stress was reported accordingly. The yield stress clearly signifies the stability of 2:1 nSi-gelatin hydrogel formulation at 25°C, 37°C, and 60°C, compared to other hydrogel concentrations. Representative images indicate dip coating of foams in different hydrogel concentrations. At all temperatures, hydrogel concentrations with high wt% of nSi-gel demonstrated very high viscosity, which made them inefficient for dip coating the foam samples. nSi-gel 2:1 showed less viscosity at all temperatures with flowability, making it suitable to dip-coat foam samples to prepare the composite, as low viscosity would be effective to surface coating as well as successful infusion of the hydrogel in the porous cavity of foam for better hemostatic ability.


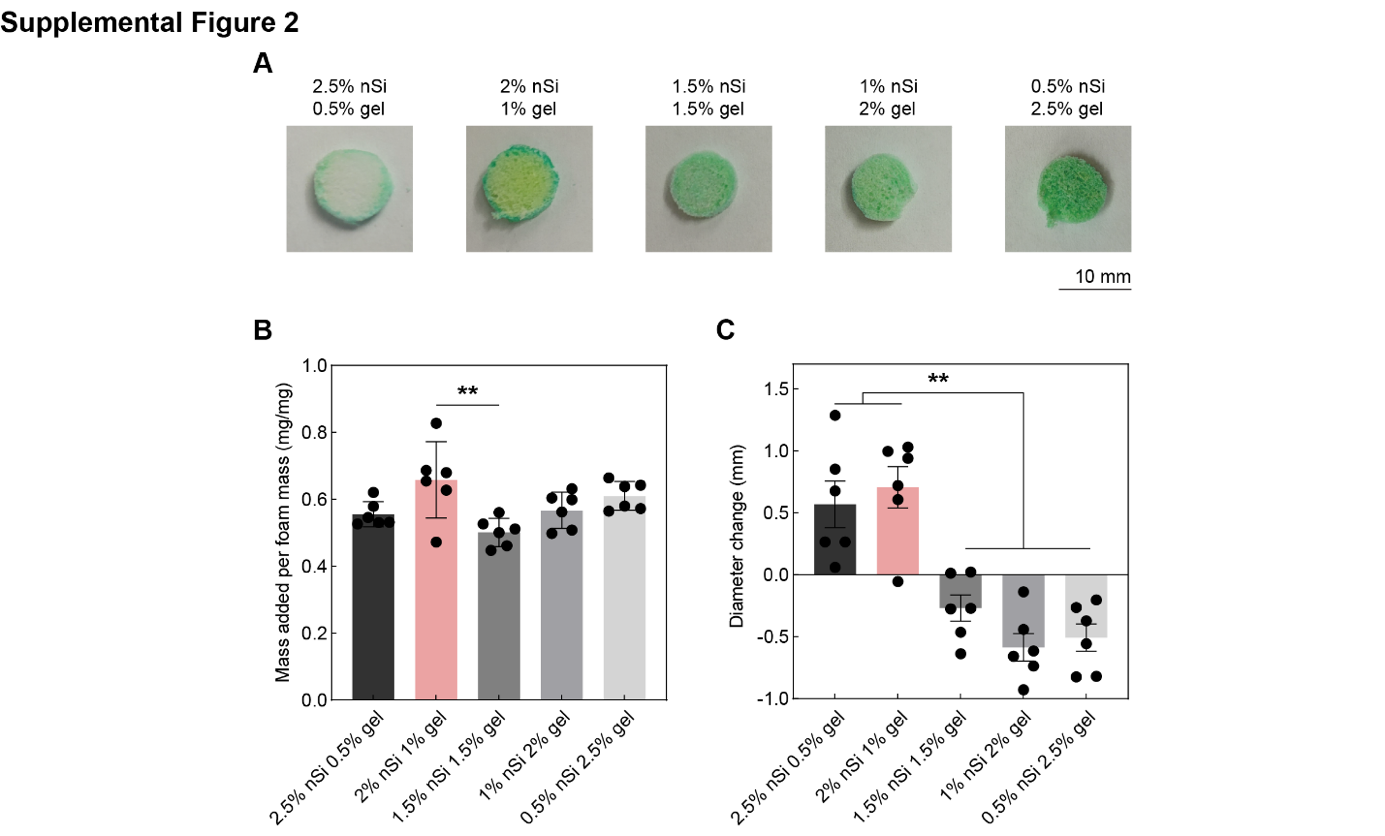


**Figure S2. Nanocomposite formulation is optimized for infiltration within the shape memory polymer foam.**

(A) Visual comparison of nanocomposite (dyed green for contrast) at different concentration ratios for nanosilicate and gelatin. Representative images are shown. nSi = nanosilicate; gel = gelatin.

(B) Analysis of the contribution of the nanocomposite to the overall mass of the composite hemostat. Results are displayed as an increase in mass per mass of foam, to represent the mass contributed by the nanocomposite relative to the initial mass of the foam. N = 6; data represented as mean ± standard deviation.

(C) Analysis of the nanocomposite’s ability to infiltrate the foam’s porous structure, measured via a change in diameter of the sample. N = 6; data represented as mean ± standard deviation.

Nanocomposites with a gelatin percentage of at least 1.5% were noted to fully infiltrate the foam; however, these samples caused a reduction in the foam’s size due to the contraction of the gelatin during drying and lyophilization processes. This was not noted in the samples prepared with nanocomposites.


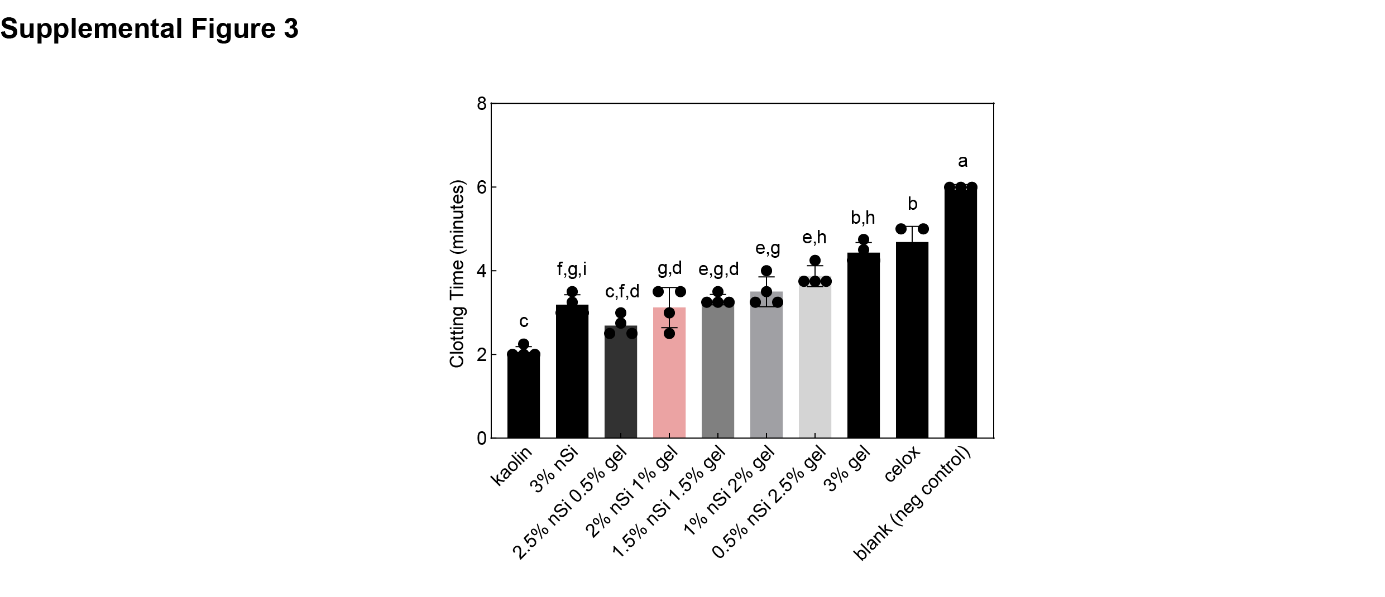


**Figure S3. Nanocomposite formulation is optimized for hemostatic ability.** Whole blood clotting time is shown for each of the various nanocomposite formulations. Clotting time was observed using an inversion test with samples observed every 15 seconds. Kaolin is used as a positive control for the nanosilicate component. 3% nanosilicate and 3% gelatin are used as extremes of the nanocomposite composition. Celox is used as a positive control for the gelatin component. A sample tube with no sample was used as a blank/negative control. Different letters indicate a statistically significant difference of at least p < 0.05. N = 3; data represented as mean ± standard deviation.


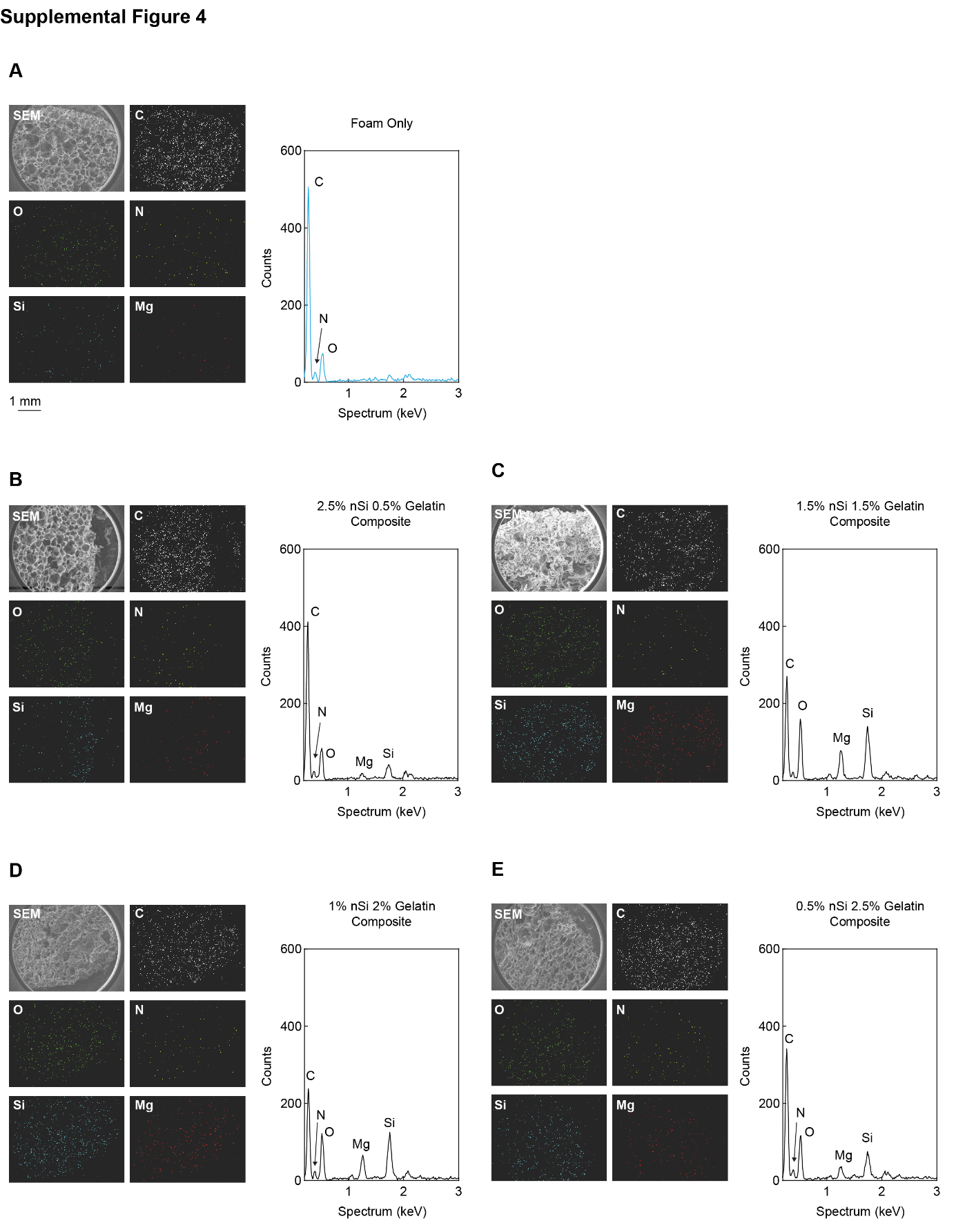


**Figure S4. Nanocomposite formulation is optimized for infiltration within the shape memory polymer foam.** Scanning electron microscopy (SEM) and energy dispersive x-ray spectroscopy (EDS) images taken samples fabricated with varying nanocomposite compositions. S4A: foam only. S4B: 2.5% nSi and 0.5% gelatin. S4C: 1.5% nSi and 1.5% gelatin. S4D: 1% nSi and 2% gelatin. S4E: 0.5% nSi and 2.5% gelatin. For spectra of 2% nSi and 1% gelatin, see Figure 1. EDS images show carbon, oxygen, nitrogen, silicon, and magnesium. The presence of silicon and magnesium indicates nanosilicate incorporation, as these elements are not found in the native foam. Representative images and spectra are shown.

**
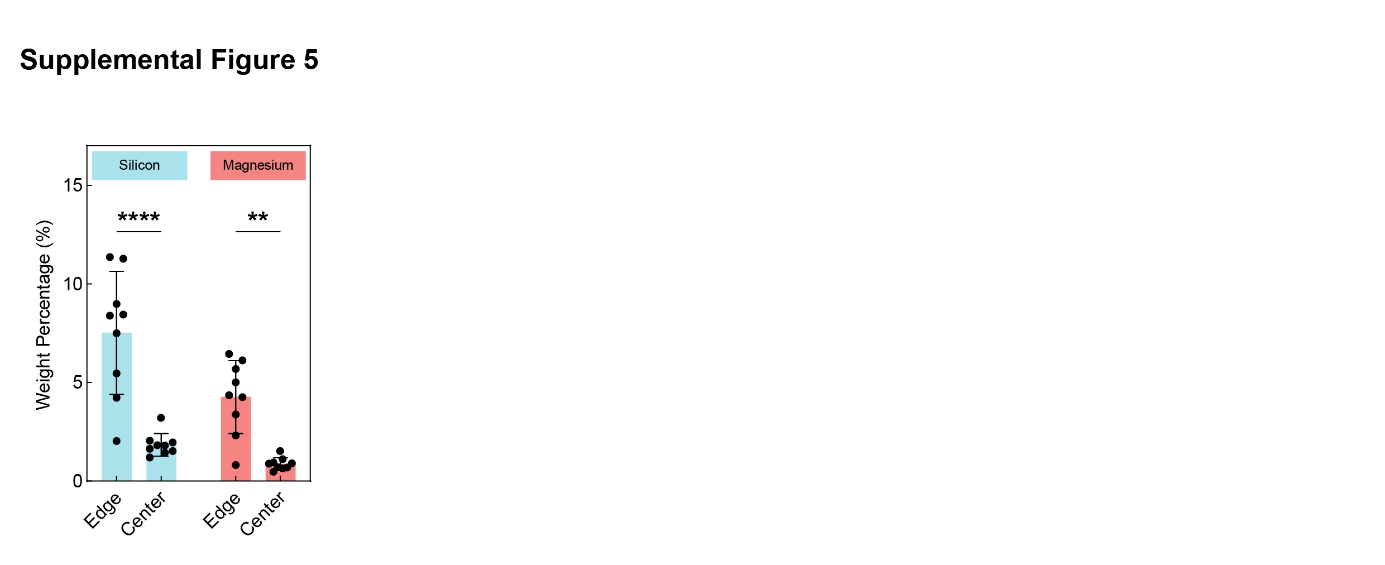
**

**Figure S5. Nanocomposite is dispersed throughout the foam with higher accumulation near the outer edge.** Energy dispersive x-ray spectroscopy (EDS) was performed to quantify the distribution of nanosilicate throughout the foam structure. Quantification was performed on three unique samples, with three edge regions and three central regions quantified per sample. N = 3 measurements per N = 3 samples; Data represented as mean ± standard deviation and analyzed via two-way Anova with Bonferroni correction.


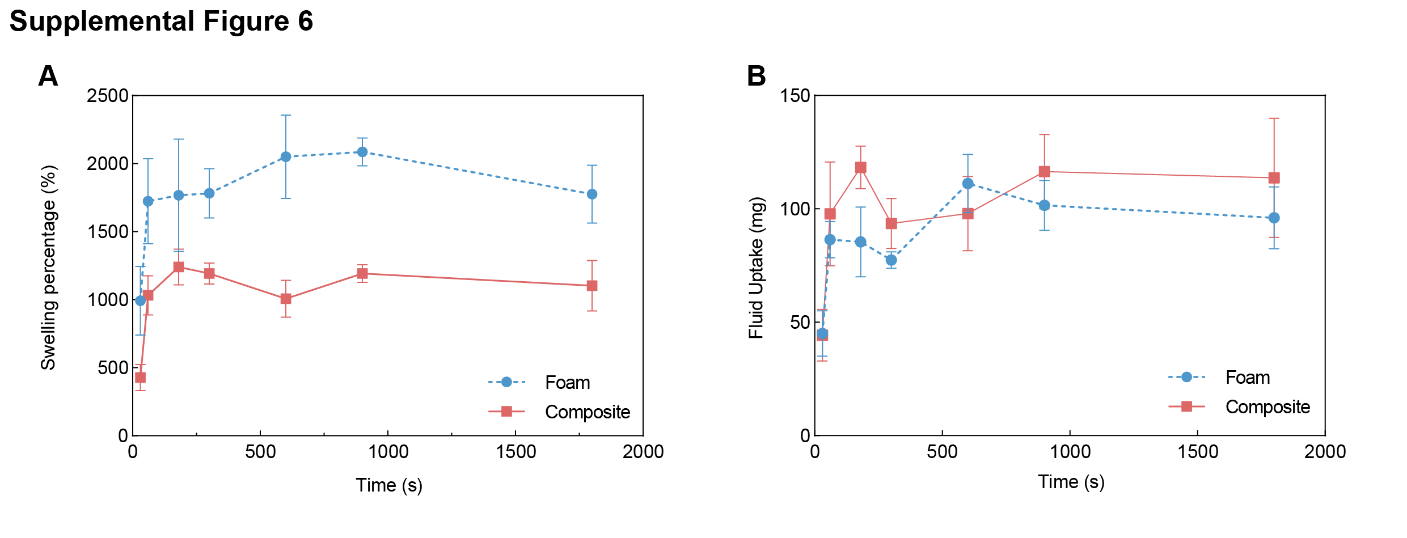


**Figure S6. Swelling percentage evaluation of foam and composite.**

(A) Quantitative swelling percentage measurement and (B) raw fluid absorptionN=3; Data represented as mean ± standard deviation.

Following compression, the swelling percentage of the foam and composite was measured in PBS under normal physiologic conditions. For foam, a rapid spike in the swelling percentage of 1723.4 ± 313.3 % was observed within 60 s and continued to increase to 2050.9 ± 306.7 % up to 600 s, which became saturated afterward whereas the swelling percentage of the composite reached 1031.3 ± 144.7% within the initial 60 s and became saturated after 600 s, exhibiting high fluid movement in the pores of the samples. Throughout the time interval, the swelling percentage of foam was observed to be higher than composite. This can be attributed to the lower mass of foam compared to the composite. When considering only the raw fluid mass absorbed by the samples, the composite demonstrated higher fluid uptake, especially at shorter time points. Although this difference is not statistically significant, this represents an improvement over our previous formulations. The presence of hydrophilic nanosilicate-gelatin hydrogel coating in the composite was able to absorb fluid rapidly within the first 60 s, which is very important for absorbing plasma rapidly to concentrate clotting factors and blood cells to treat hemorrhage.


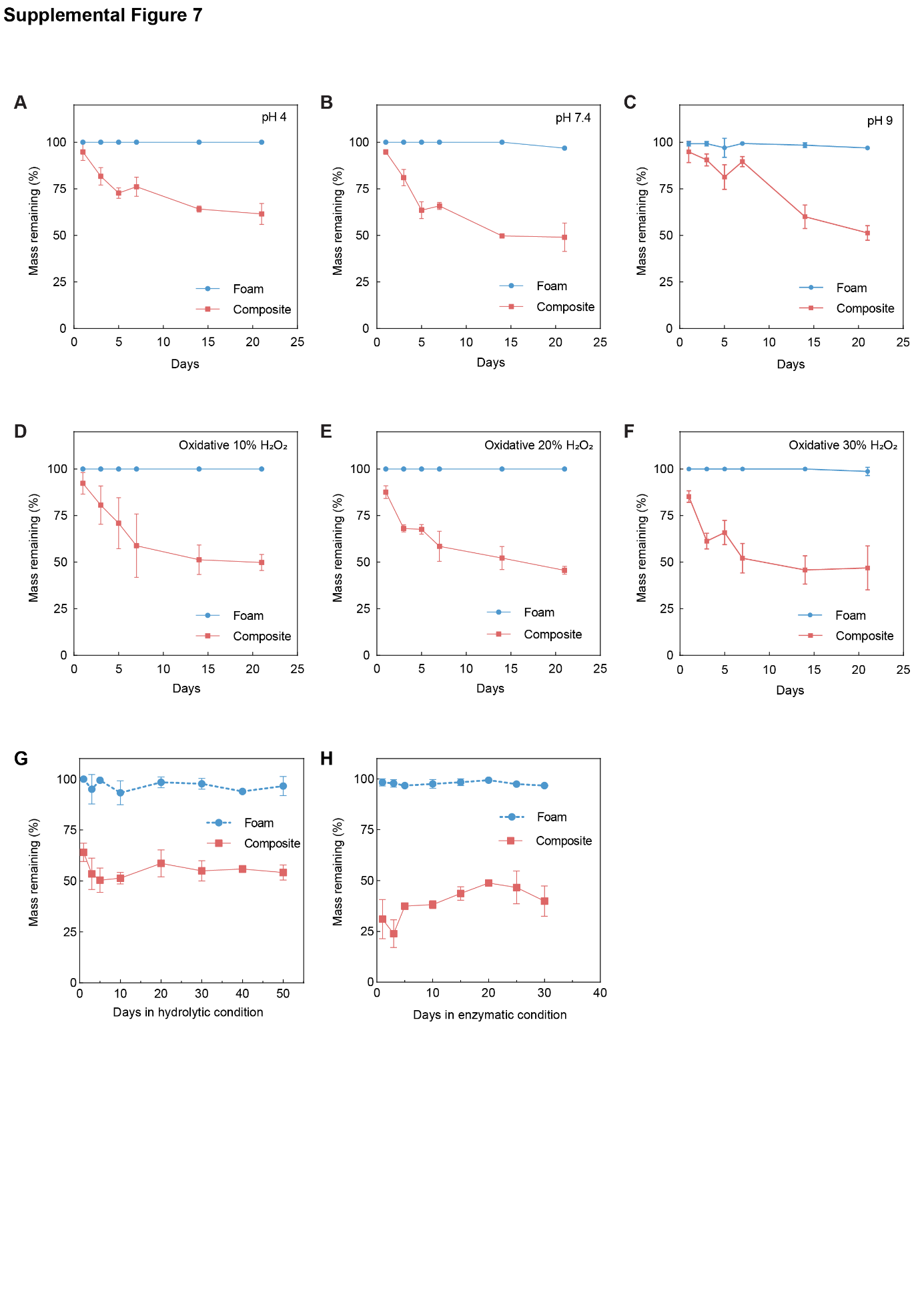


**Figure S7. Degradation evaluation of foam and composite.**

(A) Degradation study in 0.1 M acetate buffer (pH 4.0) of foam and composite showing mass loss over time for both sample types. N = 3; data represented as mean ± standard deviation.

(B) Degradation study in phosphate buffer saline (pH 7.4) of foam and composite showing mass loss over time for both sample types. N = 3; data represented as mean ± standard deviation.

(C) Degradation study in 0.1 M carbonate buffer (pH 9.0) of foam and composite showing mass loss over time for both sample types. N = 3; data represented as mean ± standard deviation.

(D) Degradation study in 10% H_2_O_2_ solutions of foam and composite showing mass loss over time for both sample types. N = 3; data represented as mean ± standard deviation.

(E) Degradation study in 20% H_2_O_2_ solutions of foam and composite showing mass loss over time for both sample types. N = 3; data represented as mean ± standard deviation

(F) Degradation study in 30% H_2_O_2_ solutions of foam and composite showing mass loss over time for both sample types. N = 3; data represented as mean ± standard deviation

(G) Accelerated hydrolytic degradation study in 0.1 N NaOH solution of foam and composite showing mass loss over time for both sample types. N = 3; data represented as mean ± standard deviation.

(H) Accelerated enzymatic degradation study in 1 mg/ml Collagenase type IV solution of foam and composite showing mass loss over time for both sample types. N = 3; data represented as mean ± standard deviation.

The results demonstrated no significant degradation of foam in any conditions for 3 weeks, whereas a trend in mass loss was observed for the composite with a gradual saturation approximately around 50%, indicating that the nanocomposite hydrogel is falling off in the initial time points, and results in mass loss, which is expected to be constant in the later time points as only the foam remains. Further, we observed sedimentation of the nanocomposite in the degradation solution and this particulate matter remained after evaporating off the degradation solution. This result is consistent with previous reports, wherein the foam remains structurally intact and starts significant degradation after 54 days due to strut fragmentation, so the obtained result is expected within 3-week time interval^1, 2^

Under hydrolytic conditions, no mass loss was observed for H40 foam for up to 50 days, indicating a low presence of ester bonds in the foam composition. Alternatively, an initial mass loss was observed for the composite with a similar trend, being observed under oxidative conditions. A remaining mass of around 50% was calculated after 30 days, which became constant throughout the 50-day time interval. This result indicates an initial diffusion of hydrogel from the composite, and post diffusion, the foam remains unaffected in hydrolytic conditions. Owing to relative hydrophobicity around the ester linkage, the lack of water penetration in the polymeric network could lead to the stability of H40 foam in a hydrolytic environment^3^. Additionally, when the degradation profile was evaluated under enzymatic conditions, no mass loss was observed for foam, but a higher mass loss of around 60% was observed within the initial 5 days, which is higher compared to oxidative and hydrolytic degradation. The remaining mass of 50% of the composites was found to be saturated after 30 days. Collagenase was able to rapidly degrade peptide bonds found in the gelatin in the composite, disrupting the polymer network for accelerated degradation at 37°C^4^. However, due to the absence of peptide bonds in the constituents of foam, the mass of foam remained unaltered after collagenase treatment.


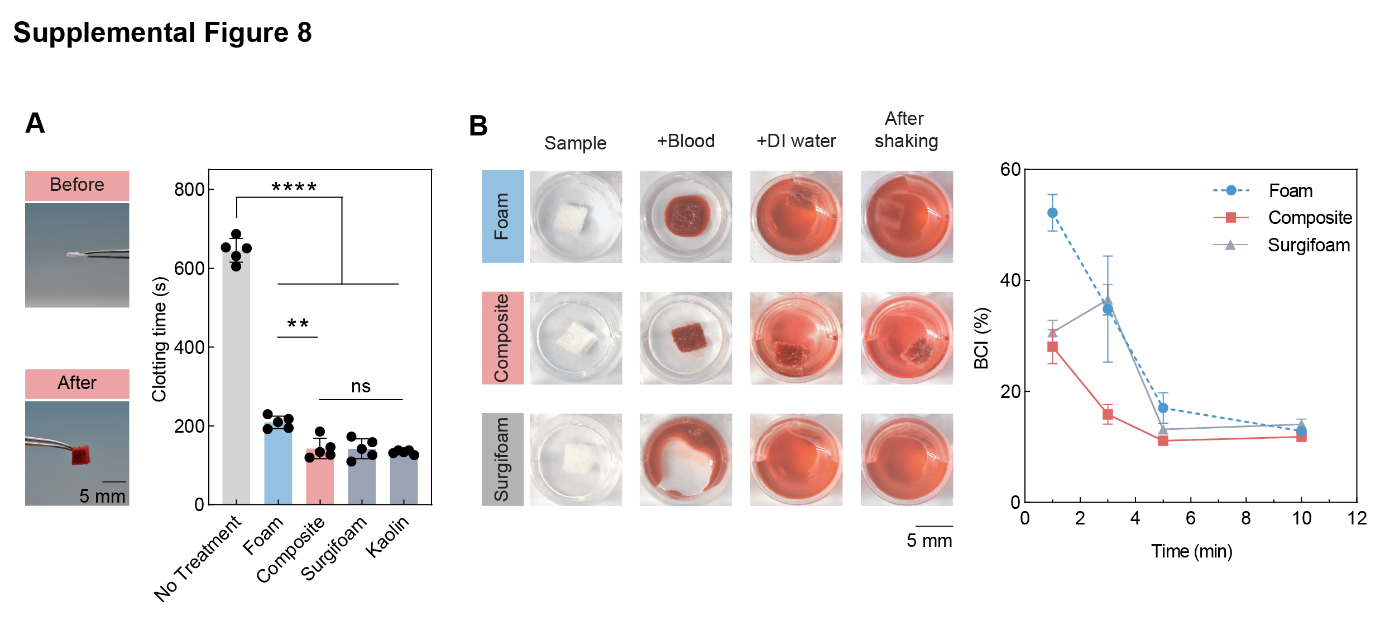


**Figure S8. *In vitro* blood clotting assessment of foam and composite on bovine blood.**

(A) Quantitative clotting time measurement determined via the inversion test. Surgifoam, a clinical product, and Kaolin serve as positive controls, and no treatment is a negative control. N = 5; data represented as mean ± standard deviation.

(B) Blood clotting index of foam, composite, and Surgifoam. Surgifoam serves as a clinical positive control. N = 3; data represented as mean ± standard deviation.

Without any treatment, the clotting time of bovine blood was calculated to be 645.4 ± 30.24 s. However, the clotting time significantly reduced when blood was added to the foam (209 ± 15.07 s) and composite (142.6 ± 26.09 s) separately. For Surgifoam and Kaolin, the clotting time was 142 ± 25.14 s and 133.2 ± 5.06 s, respectively, which were similar to the clotting time of the composite, indicating its potential to be applied in bovine blood as well.

When whole bovine blood was incubated with the samples for 1 min, the BCI of the composite was calculated to be 28.08 ± 3.05 %, whereas the BCI of foam and Surgifoam were reported to be 52.23 ± 3.32 % and 30.67 ± 2.12 %, respectively. A constant drop in BCI was observed for foam and composite after 3 min and 5 min of incubation time, where at 3 min, the BCI of foam was calculated to be 34.87 ± 9.56 and the BCI of composite further reduced to 15.85 ± 1.75 %, indicating a strong adhesion of RBC on the surface of the composite at the early time points for better coagulation. Being consistent with the BCI of human blood results, the BCI of all the experimental groups became saturated after 5 min of incubation time.


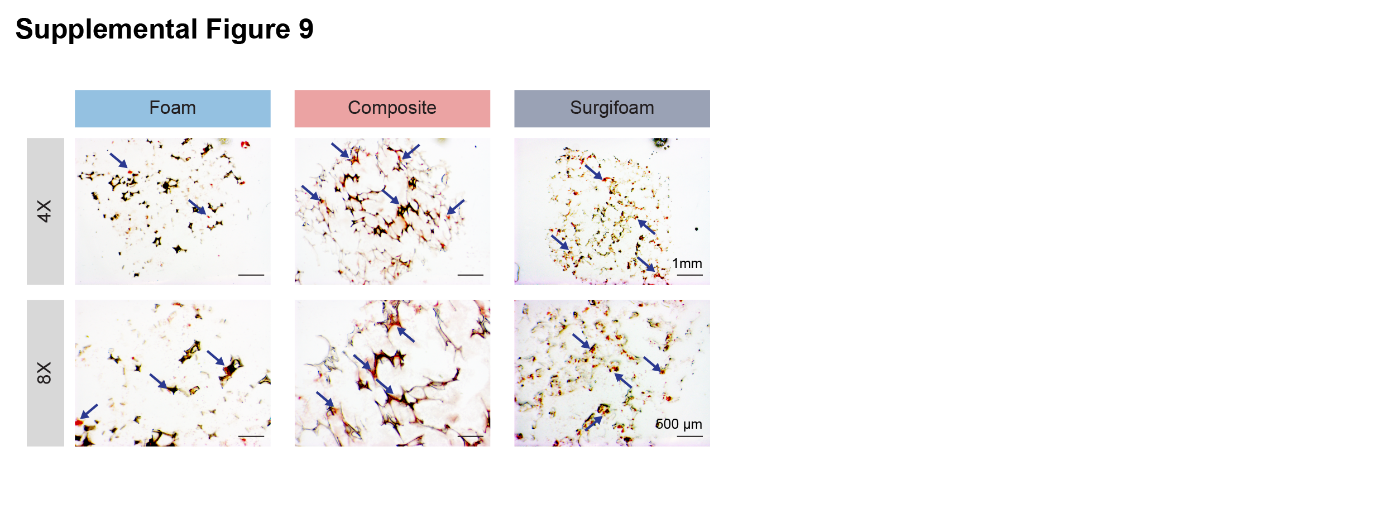


**Figure S9. Blood cell infiltration within samples.** Foam, composite, and Surgifoam were immersed in citrated whole human blood, fixed, cryopreserved, and sectioned to reveal the presence of red blood cell aggregates within the porous structure (indicated by blue arrows). All three sample types present the ability to aggregate blood cells within their porous structure. Representative images are shown. Contrast and color balance was adjusted using Adobe Photoshop; original images are available in Zenodo repository along with Photoshop files.


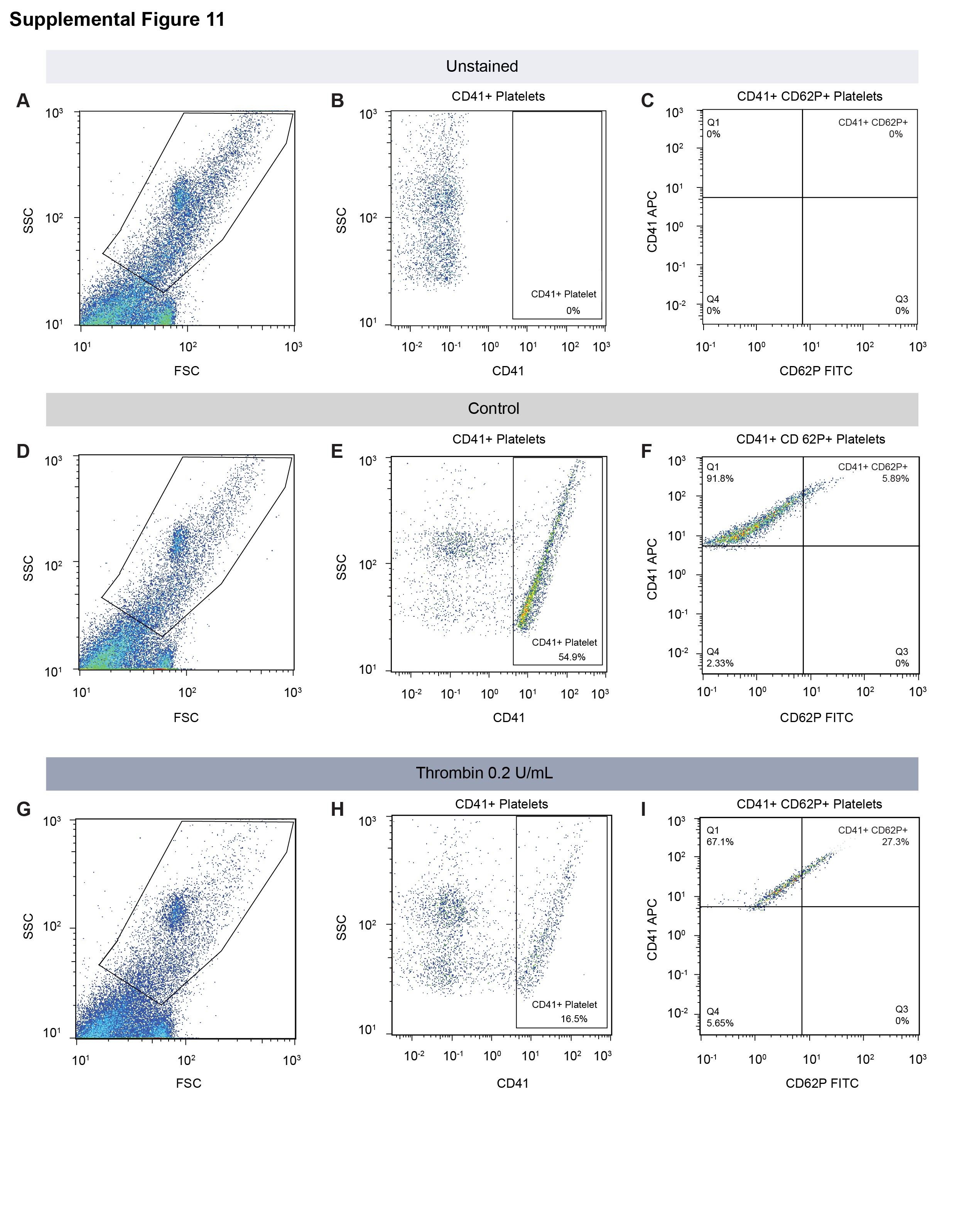


**Figure S10.** (A-I) Representative gating strategy used to identify the CD41+ platelet population for CD62 staining. Representative results are shown; replicate data is available in Zenodo repository.


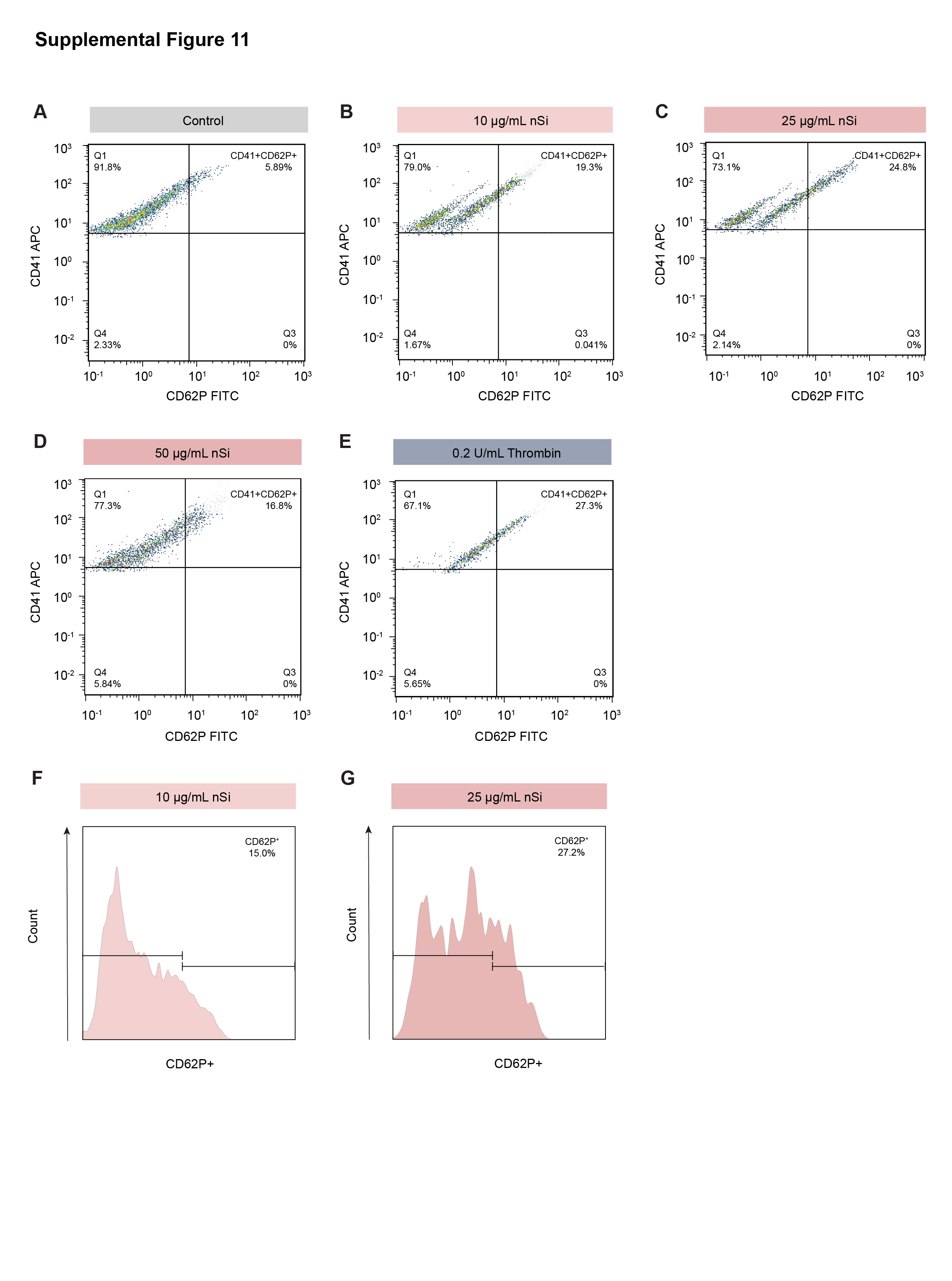


**Figure S11.** (A-E) Effects of nanosilicates on platelet activation. Representative dot plots showing the expression of CD62p on the surface of (A) unstimulated, (B) 10 µg/mL, (C) 25 µg/mL, (D) 50 µg/mL nanosilicate-treated, and (E) thrombin-treated CD41+ platelets, as measured by flow cytometry. (F, G) Representative histogram showing the expression of CD62p on the surface of (F) 10 µg/mL, and (G) 25 µg/mL nanosilicate treated CD41+ platelets, as measured by flow cytometry. Replicate data is available in Zenodo repository. Note: (A) and (E) are reproduced from Figure S10 where they are used to indicate the results of gating strategy.


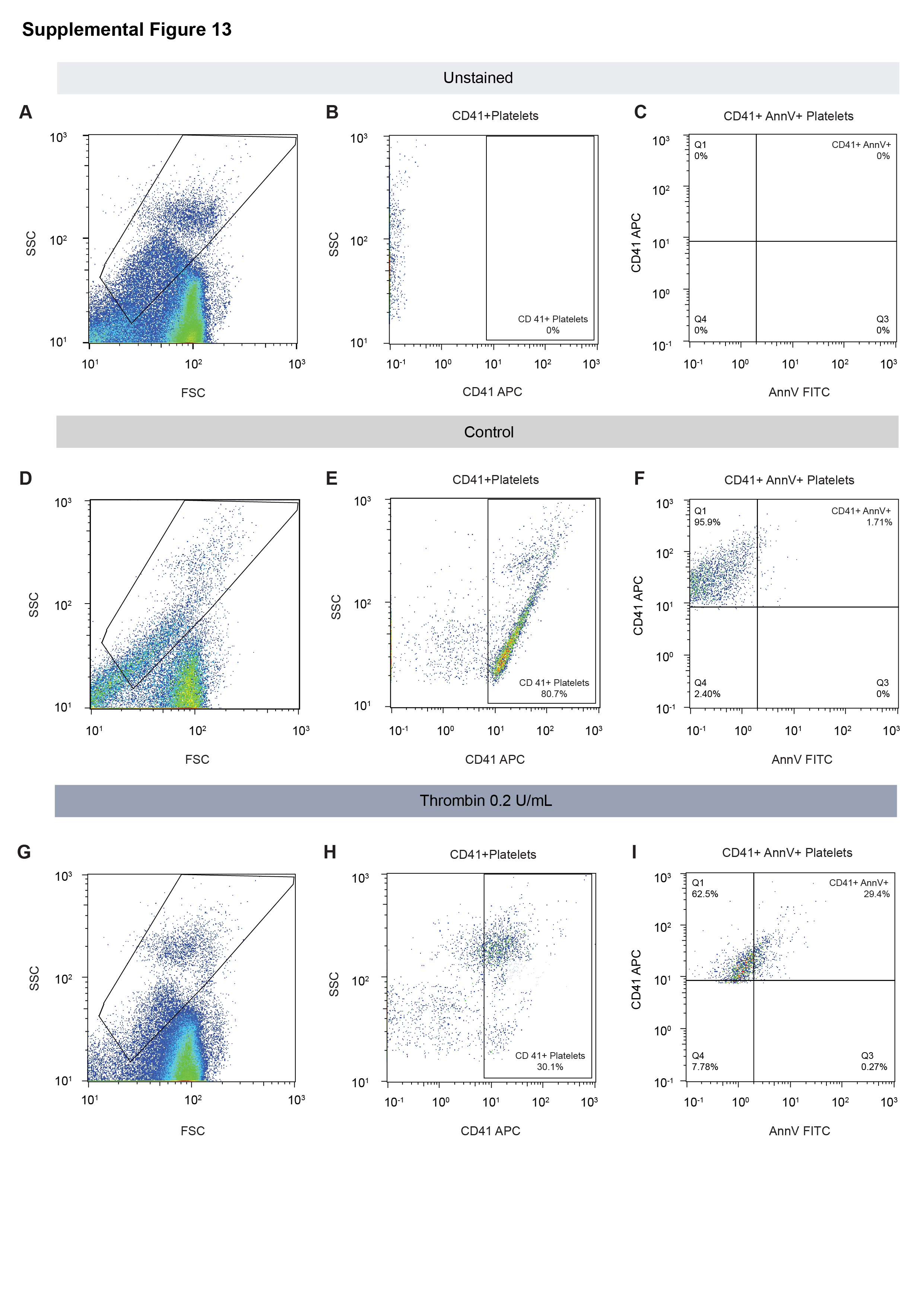


**Figure S12.** (A-I) Representative gating strategy used to identify the CD41+ platelet population for Annexin V staining. Representative results are shown; replicate data is available in Zenodo repository.


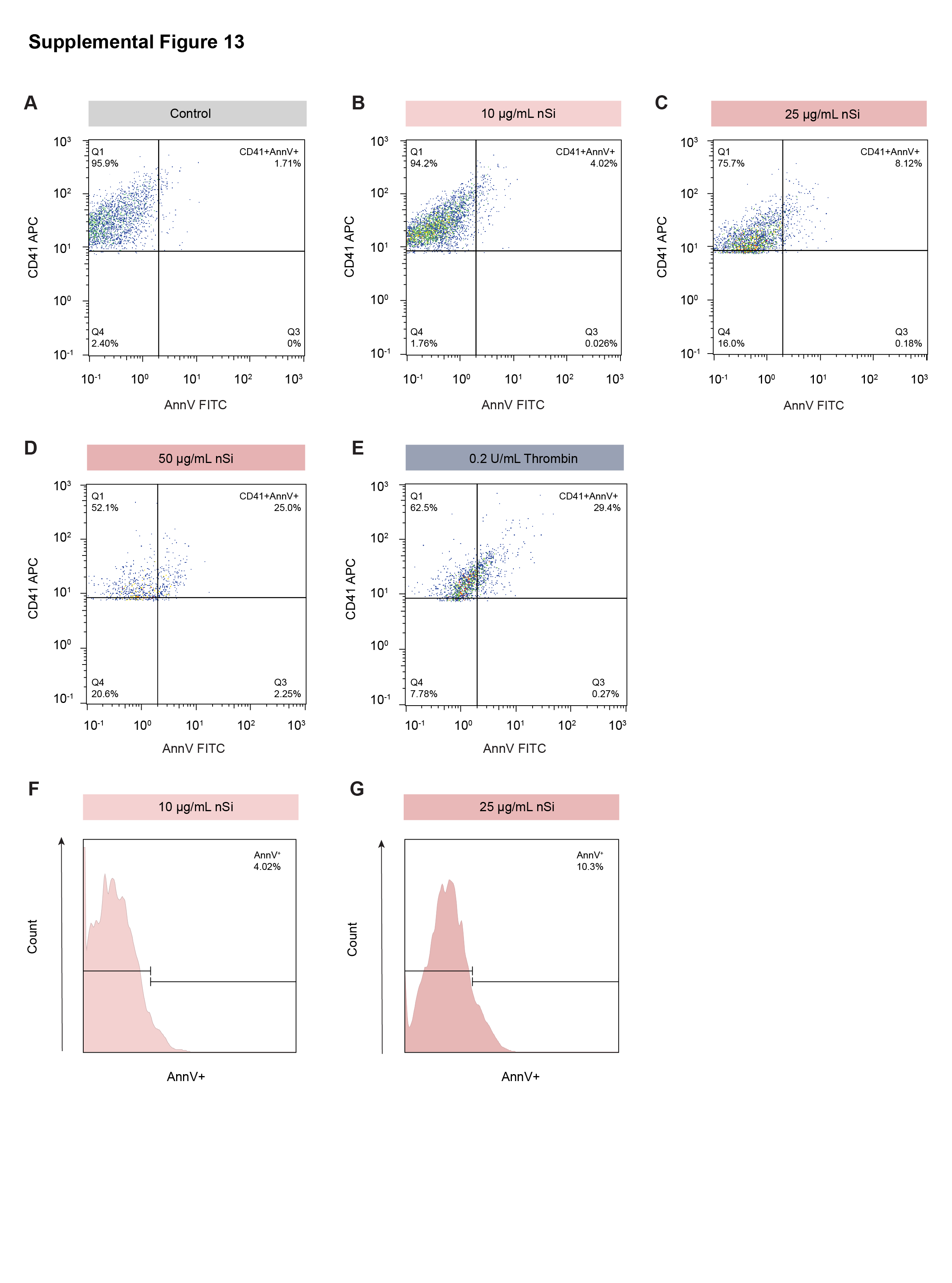


**Figure S13.** (A-G) Effects of nanosilicates on platelet activation. (A-E) Representative dot plots showing the externalization of phosphatidylserine (PS) on the surface of (A) unstimulated, (B) 10 µg/mL, (C) 25 µg/mL, (D) 50 µg/mL nanosilicate-treated, and (E) thrombin-treated CD41+ platelets, as measured by Annexin V-FITC flow cytometry. (F, G) Representative histogram showing the externalization of phosphatidylserine (PS) on the surface of (F) 10 µg/mL, and (G) 25 µg/mL nanosilicate treated CD41+ platelets, as measured by Annexin V-FITC flow cytometry. Replicate data is available in Zenodo repository. Replicate data is available in Zenodo repository. Note: (A) and (E) are reproduced from Figure S10 where they are used to indicate the results of gating strategy.


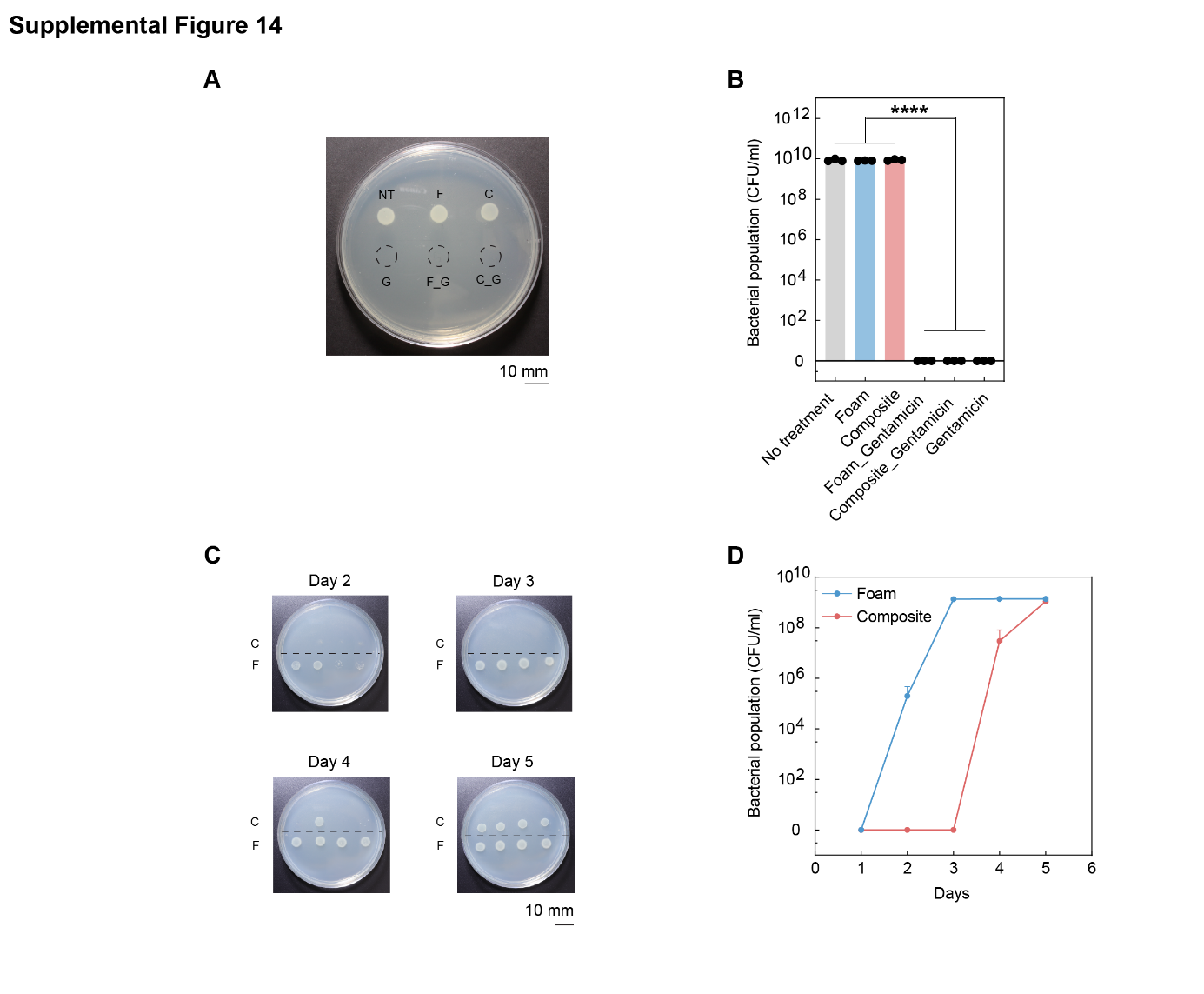


**Figure S14. Antibacterial property evaluation of foam and composite.**

(A) Representative spot test images of agar plate after 24 h culture and colony forming unit (CFU) count of viable bacterial colonies after 24 h culture after directly adding Gentamicin to the foam and the composite. NT represents no treatment; F represents foam; C represents composite; G represents Gentamicin; F_G represents gentamicin added foam; C_G represents gentamicin added composite.

(B) Quantitative CFU evaluation of spot test shown in (A). N = 3; data represented as mean ± standard deviation.

(C) Representative spot test images of agar plate after 24 h culture at different time points, after adding Gentamicin to the hydrogel precursor, while fabricating the composite. F represents foam, and C represents composite.

(D) Quantitative CFU evaluation of spot test shown in (C). N = 3; data represented as mean ± standard deviation.

The antibacterial properties of the samples and the effective release of antibacterial solution from the samples were evaluated accordingly. Without any treatment, the bacterial population was found to be 1.35 × 10^9^ ± 1.06 × 10^8^, whereas, in the presence of foam and composite, the bacterial population was calculated to be 1.48 × 10^9^ ± 1.95 × 10^8^ and 1.36 × 10^9^ ± 1.6 × 10^8^ respectively, indicating no statistical significance between the experimental groups. However, when 2 µl of Gentamicin was infused into the foam and composite, no bacterial growth was observed, which is comparable to only Gentamicin treatment. This result demonstrates the antibacterial properties of the foam and composites. When Gentamicin was added while preparing the hydrogel precursor to make the composites, the composites showed antibacterial properties for 3 days, indicating its potential as a therapeutic delivery vehicle to inhibit bacterial growth at the site of injury and accelerate wound healing, which will be explored in our future studies.


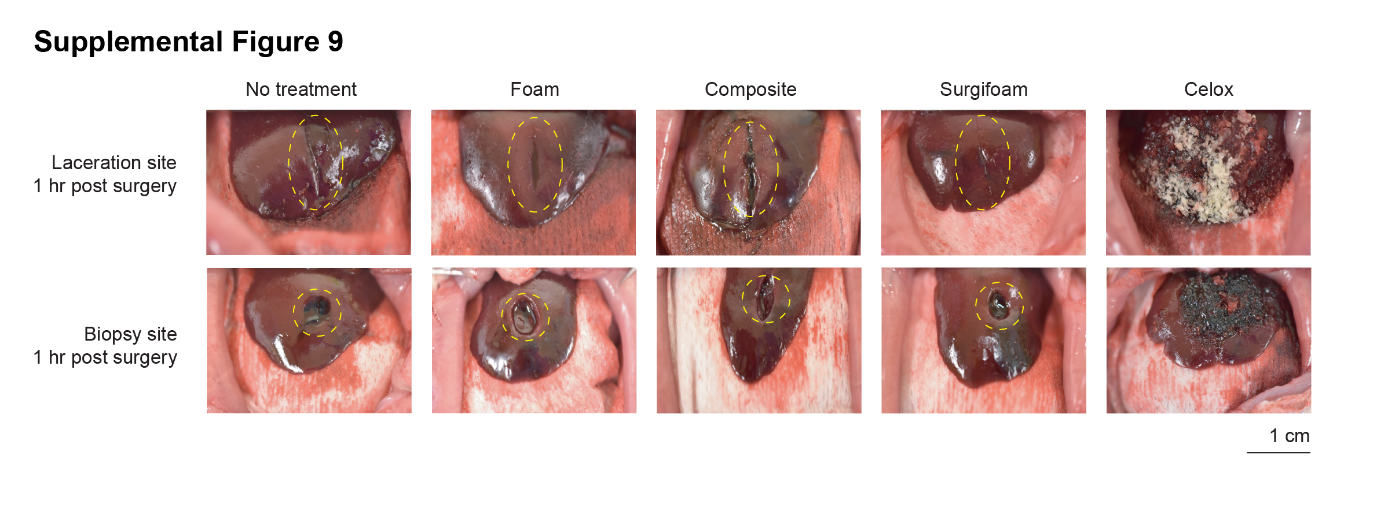


**Figure S15. Images of the biopsy punch site and lacerated liver injury site one hour post-surgery.** Representative image of sample one hour post-surgery for the no treatment group in Figure 4 is the same as the image of the laceration site one hour post-surgery for the no treatment group. Representative image of sample one hour post-surgery for the Celox group in Figure 4 is the same as the image of the laceration site one hour post-surgery for the Celox group. Representative image of sample one hour post-surgery for the no treatment group in Figure 5 is the same as the image of the biopsy punch site one hour post-surgery for the no treatment group. Representative image of sample one hour post-surgery for the Celox group in Figure 5 is the same as the image of the biopsy punch site one hour post-surgery for the Celox group.

The representative images displayed the sealing of the injured site after the sample was delivered 1 hour after surgery. No rebleeding was observed when samples were taken from the site of laceration and biopsy punch, indicating stable blood clot formation.


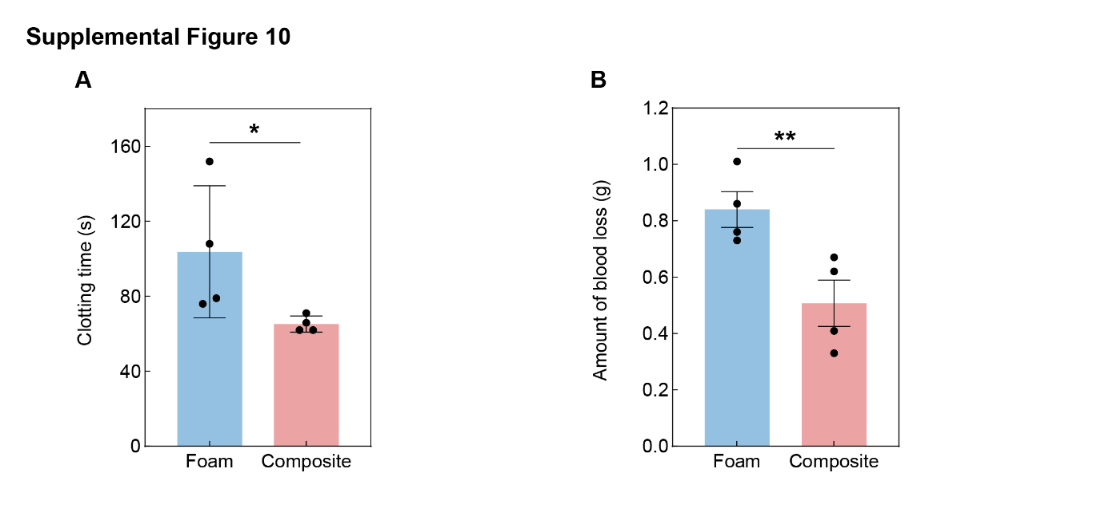


**Figure S16.** ***In vivo* rat liver biopsy punch model to evaluate composite hemostat in noncompressible hemorrhage.**

(A) Quantitative clotting time evaluation of foam and composite. N = 4, biological replicate; data represented as mean ± standard deviation.

(B) Quantitative blood loss evaluation of foam and composite. N = 4, biological replicates; data represented as mean ± standard deviation.

When clotting time and amount of blood loss between foam and composite were analyzed separately, statistical significance was observed in both cases, where composite demonstrated lower clotting time and blood loss. These results suggest that in comparison with foam, composite is more effective in a rat noncompressible hemorrhage model as well, where the reduced clotting time and blood loss could be crucial to increasing the survivability of the patients.


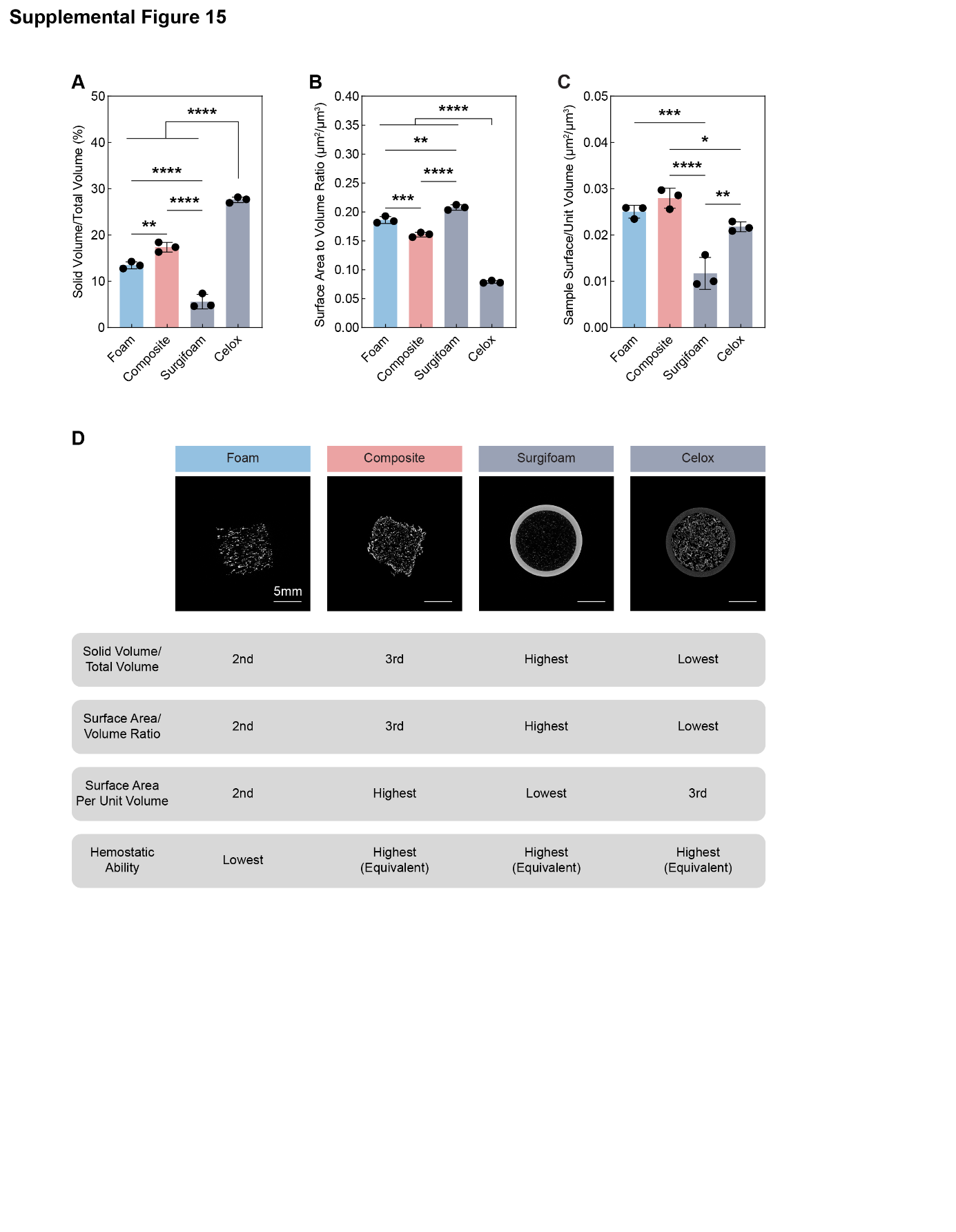


**Figure S17. MicroCT analysis of material surface area and volume fractions.**

(A) Solid volume per total volume. N = 3 non-overlapping volumes of interest; data represented as mean ± standard deviation.

(B) Surface area to volume ratio. N = 3 non-overlapping volumes of interest; data represented as mean ± standard deviation.

(C) Sample surface area per unit volume. N = 3 non-overlapping volumes of interest; data represented as mean ± standard deviation.

(D) Overview of trends concerning hemostatic ability, surface area, and volume fractions. Representative slices of CT scan reconstruction are shown. Note that the rings around Surgifoam and Celox samples are sample holders to prevent the sample from spilling or melting during CT imaging.

Analysis of the volume fraction, surface area to volume ratio, and surface area per unit volume of the foam, composite, Surgifoam, and Celox revealed trends distinct from the trends observed regarding the hemostatic ability of these materials. These results suggest that the hemostatic ability is not solely due to the porosity or density of the material and instead requires consideration of the surface activity of the material.

**
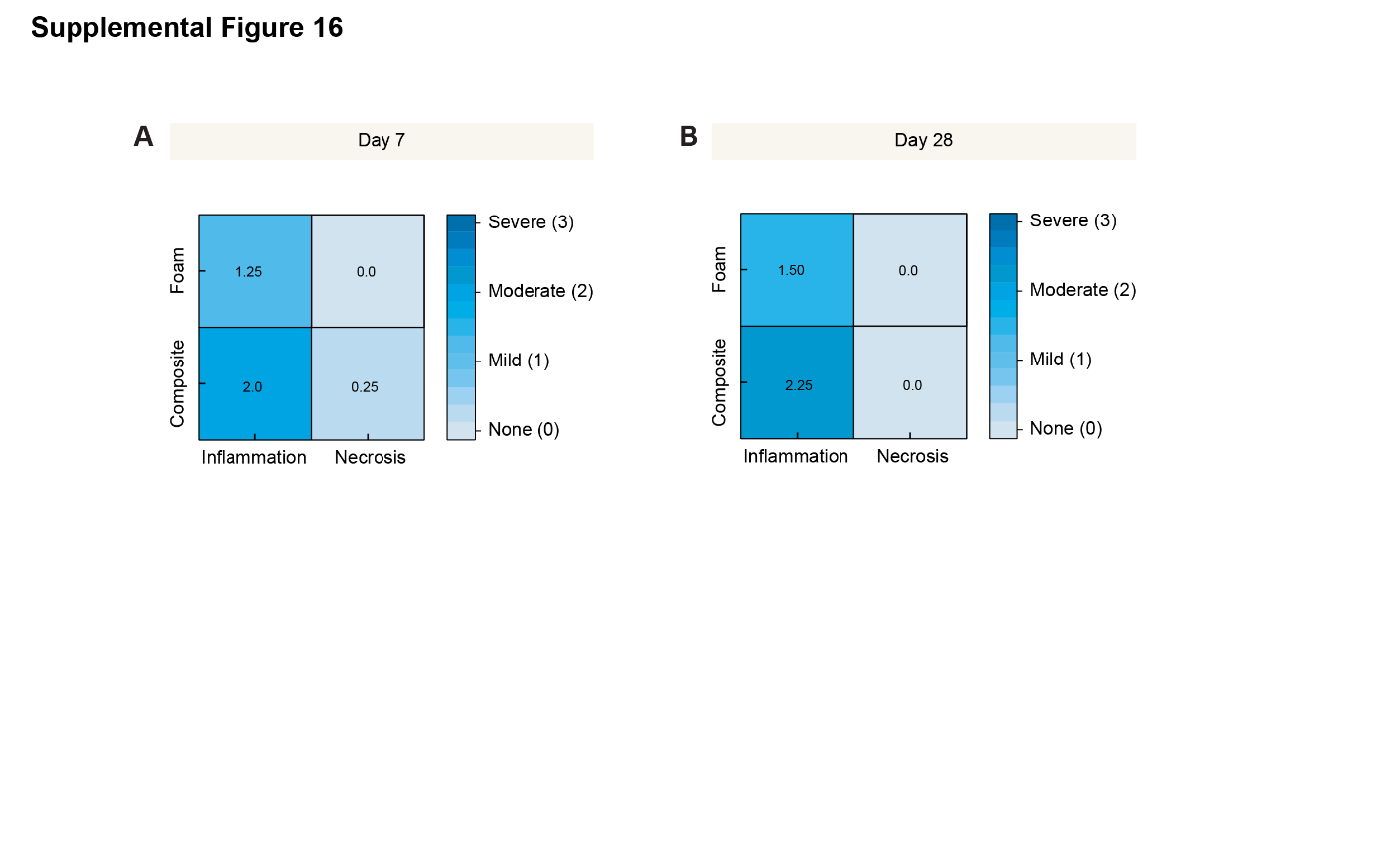
**

**Figure S18. Pathology scoring of histology sections for inflammation and necrosis.**

(A) Heat map displaying subjective histology scores from an expert pathologist. The score is for necrosis and inflammation localized around the injury site as visualized in the histology of excised tissue, 7 days post-surgery. N = 4; data represented as mean.

(B) Heat map displaying subjective histology scores from an expert pathologist. The score is for necrosis and inflammation localized around the injury site as visualized in the histology of excised tissue, 28 days post-surgery. N = 4; data represented as mean.

After 7 days of implantation, no-minimal necrosis was observed for composite and foam, with mild-moderate inflammation, whereas after 28 days,no necrotic cells were found out for both the samples around the site of implantation, but a minimum increase in inflammation was observed for both the samples. This response is expected in this time duration, which is expected to resolved in the later time points^5-8^.


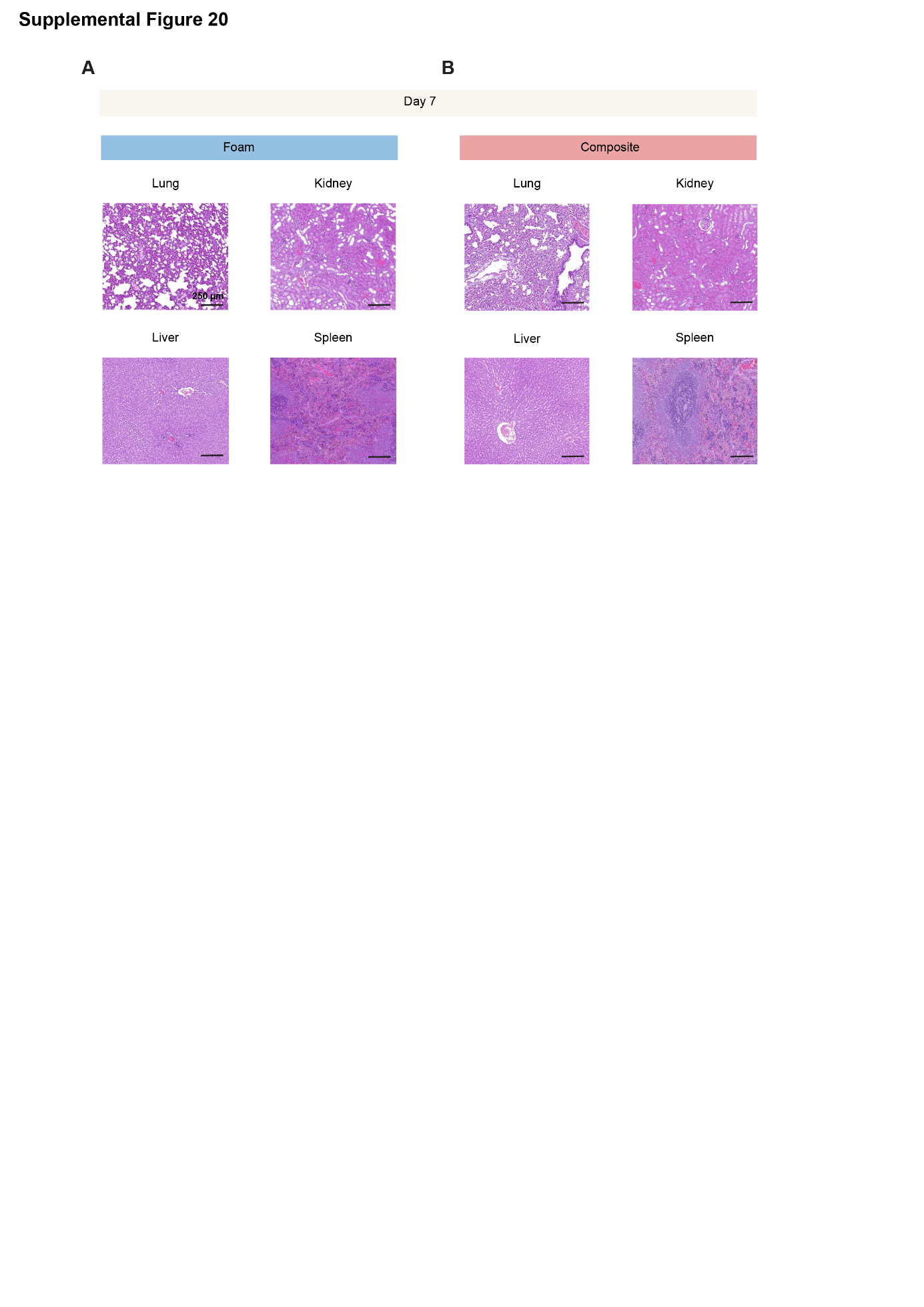


**Figure S19. Histological analysis of organs after 7 days of subcutaneous implantation**

1. Representative H&E staining of various major organs of rats, implanted with foam.
2. Representative H&E staining of various major organs of rats, implanted with composite.

The histological analysis demonstrated no observable change in cellular morphology in any of the major organs of animals after 7 days, implanted with both foam and composite, indicating no significant systemic inflammation.

**Supplemental Video 1. Ex vivo porcine liver biopsy punch.**

A porcine liver was obtained through the Texas A&M University tissue share program. The liver was harvested from the animal immediately following euthanasia and used within 4 hours of collection. The liver was placed under a heat lamp to maintain the temperature of the tissue at approximately 37°C. A 6-mm biopsy punch was used to create a cavity in the tissue. Bleeding was observed from the cavity. Compressed composite samples were injected into the cavity using a 1-ml syringe. The syringe had the end cut off to facilitate sample extrusion. Samples were allowed to expand within the cavity and then removed. Upon removal, it was apparent that the samples had expanded and had absorbed blood within the wound cavity. Approximately 0.3 mL of compressed samples were injected into the wound, representing 7 composites corresponding to approximately 0.9 mL of expanded samples. This result shows that we can achieve approximately 3x volume filling of a cavity with the composites, which indicates the potent application of the composite in the large animal model.

**REFERENCES**

(1) Jang, L. K.; Fletcher, G. K.; Monroe, M. B. B.; Maitland, D. J. Biodegradable shape memory polymer foams with appropriate thermal properties for hemostatic applications. *Journal of Biomedical Materials Research Part A* **2020**, *108* (6), 1281-1294.

(2) Hargett, S. E.; Lokhande, G. K.; Duran, J.; Hirani, Z.; Jang, L. K.; Foster, S.; Deo, K. A.; George, S.; Javed, M.; Ware, T. H. Nanoengineered Shape‐Memory Hemostat. *Small Science* **2025**, *5* (2), 2400321.

(3) Vakil, A. U.; Petryk, N. M.; Shepherd, E.; Beaman, H. T.; Ganesh, P. S.; Dong, K. S.; Monroe, M. B. B. Shape Memory Polymer Foams with Tunable Degradation Profiles. *ACS Appl Bio Mater* **2021**, *4* (9), 6769-6779. DOI: 10.1021/acsabm.1c00516 From NLM Medline.

(4) Saito, T.; Tabata, Y. Preparation of gelatin hydrogels incorporating low-molecular-weight heparin for anti-fibrotic therapy. *Acta biomaterialia* **2012**, *8* (2), 646-652.

(5) Gaharwar, A. K.; Avery, R. K.; Assmann, A.; Paul, A.; McKinley, G. H.; Khademhosseini, A.; Olsen, B. D. Shear-thinning nanocomposite hydrogels for the treatment of hemorrhage. *ACS nano* **2014**, *8* (10), 9833-9842.

(6) Avery, R. K.; Albadawi, H.; Akbari, M.; Zhang, Y. S.; Duggan, M. J.; Sahani, D. V.; Olsen, B. D.; Khademhosseini, A.; Oklu, R. An injectable shear-thinning biomaterial for endovascular embolization. *Science translational medicine* **2016**, *8* (365), 365ra156-365ra156.

(7) Herting, S. M.; Ding, Y.; Boyle, A. J.; Dai, D.; Nash, L. D.; Asnafi, S.; Jakaitis, D. R.; Johnson, C. R.; Graul, L. M.; Yeh, C. In vivo comparison of shape memory polymer foam‐coated and bare metal coils for aneurysm occlusion in the rabbit elastase model. *Journal of Biomedical Materials Research Part B: Applied Biomaterials* **2019**, *107* (8), 2466-2475.

(8) Rhee, J. Y.; Trocciola, S. M.; Dayal, R.; Lin, S.; Chaer, R.; Kumar, N.; Mousa, A.; Bernheim, J.; Christos, P.; Prince, M. Treatment of type II endoleaks with a novel polyurethane thrombogenic foam: induction of endoleak thrombosis and elimination of intra-aneurysmal pressure in the canine model. *Journal of vascular surgery* **2005**, *42* (2), 321-328.
